# Performance Profiles of Short DNA Barcode Segments for Family Level Detection of Asteraceae Within Asterales

**DOI:** 10.3390/plants15172736

**Published:** 2026-09-07

**Authors:** Shuai Jiang, Shunxing Ye, Yian Wang, Hongyi Zhu, Yangxu Wu, Zeqi Li, Junnan Hang, Xiaoping Li, Yan Yin, Qinghua Lu, Xinhong Guo

**Affiliations:** 1 College of Biology, Hunan University, Changsha 410082, China; gs2022@hnu.edu.cn (S.J.); wya@hnu.edu.cn (Y.W.); zhuhy0623@hnu.edu.cn (H.Z.); wuyangxu@hnu.edu.cn (Y.W.); lizegi2023@hnu.edu.cn (Z.L.); hangjunnan@hnu.edu.cn (J.H.); bioxpli@hnu.edu.cn (X.L.); yinyan@hnu.edu.cn (Y.Y.); 2 Yuelushan Laboratory, Changsha 410082, China; 3 Hunan Research Center of the Basic Discipline for Cell Signaling, College of Biology, Hunan University, Changsha 410082, China; 4 Institute of Brain Science and Translational Research, Fudan University, Shanghai 200032, China; sxyefudan@163.com

**Keywords:** Asteraceae, DNA barcoding, family-level discrimination, taxonomic differentiation

## Abstract

Short DNA barcodes may facilitate sequence recovery from degraded material, but their ability to retain target-family identity while excluding related taxa varies among genomic regions. We computationally evaluated 16 nuclear, plastid, and mitochondrial marker regions from 11 Asterales families using 279,956 NCBI locus–record matches and an accession-disjoint discovery/test design. Thirty-one candidate segments of 50–200 bp (mean, 98.55 bp) were screened in discovery data and evaluated for within-Asteraceae sequence recall, differentiation from non-Asteraceae Asterales, in silico primer behavior, phylogenetic placement, and exploratory matching across 808 metadata-defined metagenomic samples. Conserved regions such as *matR* and *rbcL* showed high within-Asteraceae identity, whereas *ITS1*, *ITS*, and *trnH*-*psbA* showed larger differences from related-family backgrounds; *ITS2* and *ycf1* showed intermediate profiles. Candidate segments were placed within or immediately adjacent to Asteraceae reference branches in segment-specific maximum-likelihood analyses, although support and topology varied among regions. Metadata-defined target-containing groups had higher mean query coverage and identity than background groups; because target presence was not independently verified and no classifier was fitted, these comparisons were descriptive and did not estimate diagnostic accuracy. Definitionally linked sequence statistics were interpreted as structural associations rather than evidence of causal evolutionary mechanisms. These results provide a family-level computational comparison of candidate short segments for Asteraceae detection within Asterales. Species identification, operational marker combinations, threshold robustness, and laboratory performance require validation using taxonomically dense, voucher-linked, and experimentally characterized datasets.

## 1. Introduction

Accurate, rapid, and reproducible species identification was fundamental to plant-resource regulation, ecological monitoring, analysis of complex environmental samples, and traceability of medicinal and edible plants [1,2,3,4]. However, plant DNA barcoding had long faced a fundamental conflict: loci that could be stably amplified and recovered across broad taxonomic groups were often not those with the highest species-level resolution, whereas highly variable regions rich in taxonomic information were frequently constrained by primer universality, segment length, and lineage specificity. Accordingly, the CBOL Plant Working Group designated *rbcL* and *matK* as core barcodes for land plants while also acknowledging that no single locus could simultaneously satisfy the ideal criteria of universal amplification, high recovery, and high resolution [5]. Subsequent large-scale studies promoted the inclusion of *ITS* and *ITS2* in the core plant barcode system [6,7], and *ycf1* was proposed as a chloroplast candidate locus with higher resolution [8]. Together, these advances indicated that plant barcoding had never been a fully resolved standardization problem, but rather had remained a problem of how to achieve an optimal compromise among different objectives.

This conflict was particularly pronounced in plants because the performance of candidate regions depended not only on the number of variable sites but also on genomic compartment, mode of inheritance, and lineage history. Chloroplast regions generally provided favorable sequence recoverability and compatibility with reference databases, but their relatively conservative evolutionary rates and potential plastid introgression could reduce resolution among closely related species [9,10]. Ribosomal DNA spacer regions often accumulated more interspecific differences but could be affected by incomplete homogenization among copies, hybridization, and lineage admixture [11]. Therefore, evaluation of candidate regions should not have been limited to the degree of variation but should also have considered stability within the target taxon, the ability to exclude closely related backgrounds, and practical detectability.

Asteraceae and the order Asterales provided a challenging system for evaluating family-level target recall and related-background exclusion. Asteraceae is species rich and phylogenetically complex, with cytonuclear discordance, rapid diversification, hybridization, and introgression reported in nuclear and plastid studies [12,13,14,15]. Consequently, a segment that distinguishes Asteraceae from other Asterales families cannot, without taxonomically dense congeneric testing, be assumed to identify species within Asteraceae.

Plant-identification applications increasingly include degraded DNA, mixed backgrounds, low-abundance targets, and environmental metagenomic samples. Shorter markers can improve recovery from fragmented templates but may contain less taxonomic information [16,17,18,19]. Existing work has often emphasized a small number of standard loci or idealized reference matching. Here, public sequences and metadata-defined metagenomes were used to compare several aspects of candidate-segment behavior. Because neither wet-laboratory amplification nor independently verified presence/absence was available, these analyses were treated as computational screening and exploratory external matching rather than experimental or diagnostic validation.

Accordingly, this study evaluated 16 candidate regions across 11 families of Asterales and screened short DNA barcode segments of 50–200 bp. The analyses asked two bounded questions: (i) how conservation, within-Asteraceae recall, and exclusion of non-Asteraceae Asterales backgrounds varied among regions; and (ii) whether the resulting multidimensional profiles could generate hypotheses for task-specific marker selection. The study did not test species identification within Asteraceae and did not establish operational screening/confirmation rules, clinical-style thresholds, sensitivity, or specificity.

## 2. Results

### 2.1. Patterns of Genetic Variation Across Candidate Regions in Asteraceae and Asterales

**Figure 1:**
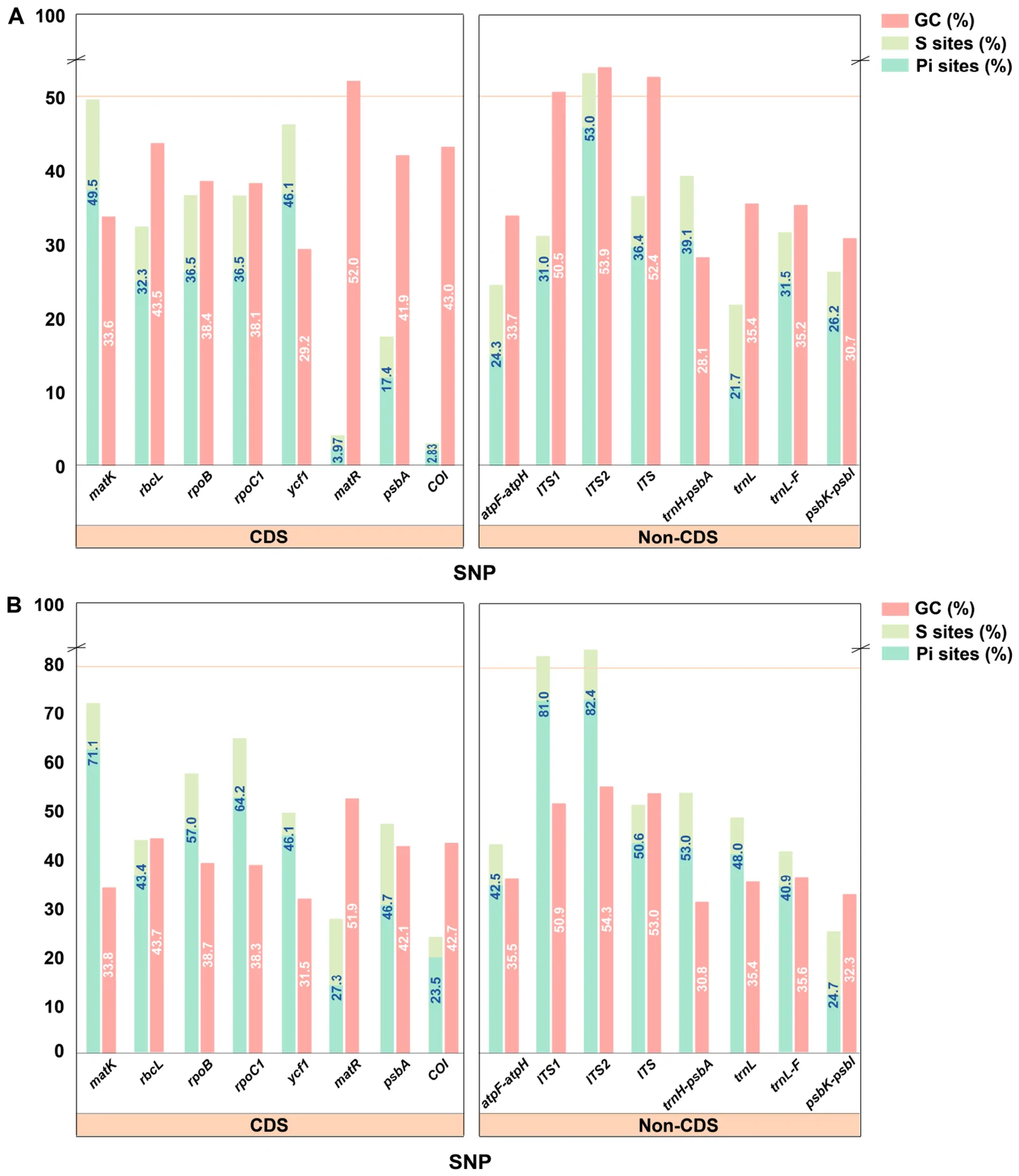
Comparison of SNP-related characteristics among selected CDS and Non-CDS gene regions in Asteraceae (**A**) and Asterales (**B**) plants.

For most candidate regions, expanding the comparison level from Asteraceae (Figure 1A) to Asterales (Figure 1B) markedly increased the proportions of variable and parsimony-informative sites (Pi sites), whereas GC content changed relatively little. In the non-coding sequences (Non-CDSs), *ITS1* and *ITS2*, the proportions of Pi sites reached 81.0% and 82.4%, respectively, in Asterales, exceeding the corresponding values of 31.0% and 53.0% in Asteraceae; *trnH-psbA* likewise showed high information content. Among the coding sequences (CDSs), *matK*, *ycf1*, and *rpoC1* showed relatively high variation, whereas *matR* and *COI* remained comparatively conserved at both taxonomic levels. The GC content of *matR* was approximately 52%, but its proportion of Pi sites was low, indicating that GC composition did not have a simple correspondence with sequence polymorphism.

**Figure 2:**
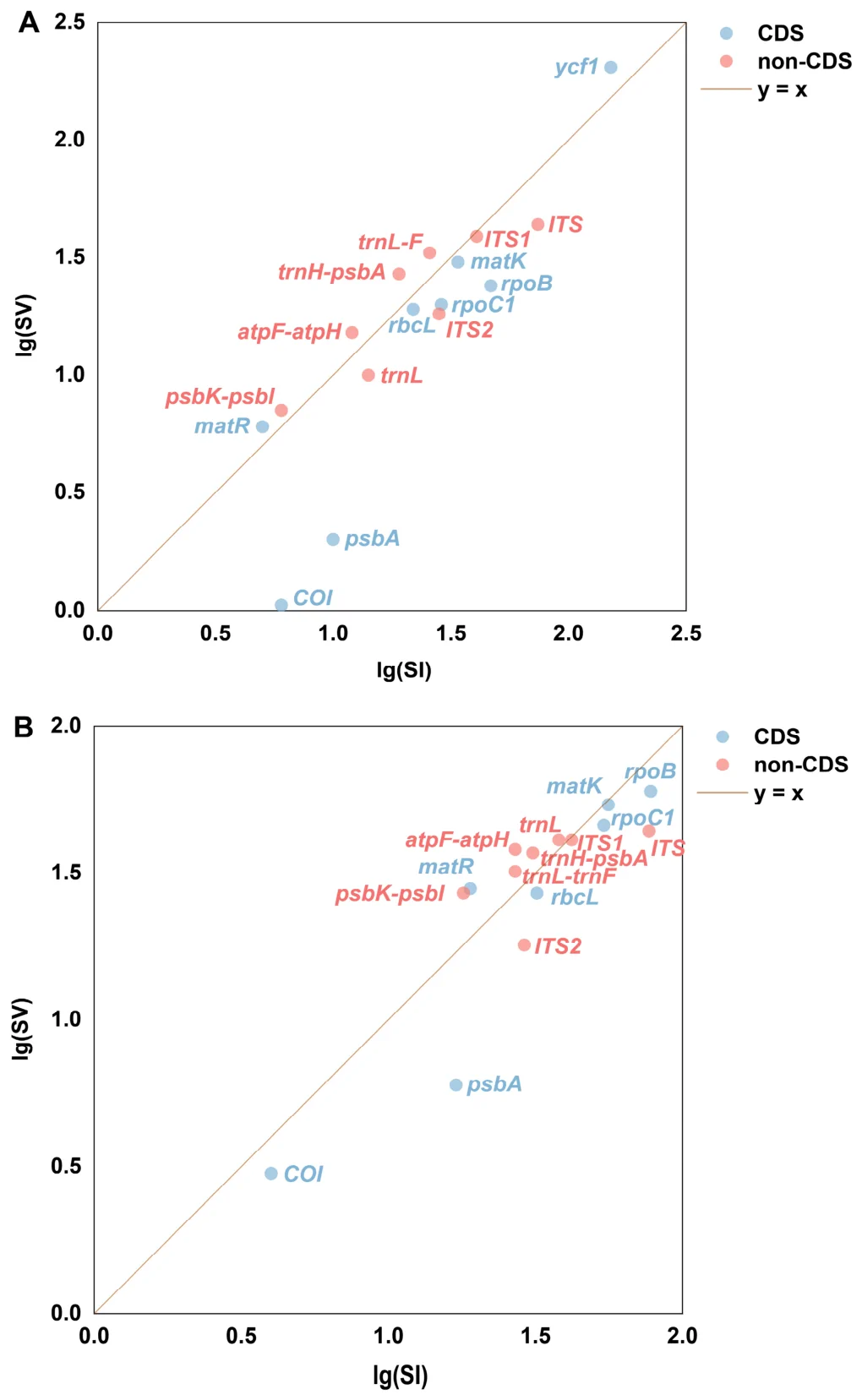
Logarithmic distributions of nucleotide transition and transversion frequencies in selected CDS and Non-CDS regions of Asteraceae (**A**) and Asterales (**B**) plants. Note: The x-axis represented the logarithm of the nucleotide transition frequency, lg(SI), whereas the y-axis represented the logarithm of the nucleotide transversion frequency, lg(SV). Blue dots represented CDSs, whereas red dots represented Non-CDSs. The diagonal line, y = x, indicated equal transition and transversion frequencies. Data points located above the diagonal indicated SV > SI, whereas those located below the diagonal indicated SI > SV. Because logarithmic coordinates were used, a difference of 1 between coordinates corresponded approximately to a 10-fold difference in the original frequencies. Therefore, the distances among different loci in the plot reflected not only directional differences but also orders-of-magnitude differences in substitution intensity. *ITS*, *ITS1*, and *ITS2* were nuclear genomic markers; *COI* and *matR* were mitochondrial markers; and most of the remaining regions were chloroplast coding genes or chloroplast non-coding intergenic regions.

The numbers of transitions and transversions differed markedly among candidate regions (Figure 2). Overall, Non-CDSs accumulated more substitutions than most CDSs. Within Asteraceae (Figure 2A), *ITS*, *ITS1*, and *ycf1* showed relatively high substitution levels, whereas *COI* and *psbA* showed relatively low levels. Most candidate regions showed higher substitution counts at the Asterales level (Figure 2B); however, transition and transversion counts alone were insufficient to determine substitution saturation and were therefore treated as descriptive evidence of differences in evolutionary rates among regions.

**Figure 3:**
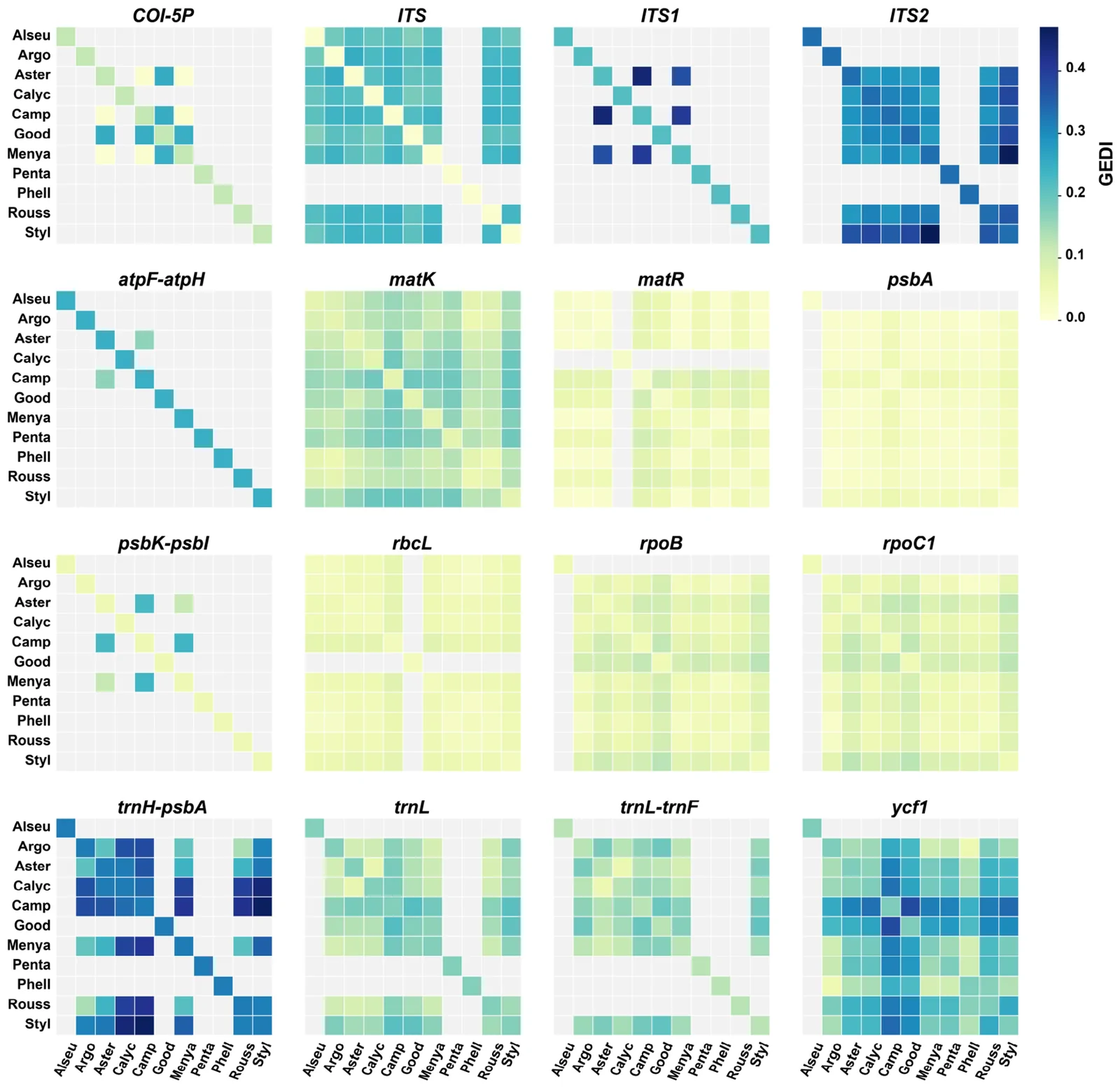
Family level GEDI matrix for Asterales plants based on 16 candidate gene regions. The horizontal and vertical axes represented Alseuosmiaceae (Alseu), Argophyllaceae (Argo), Asteraceae (Aster), Calyceraceae (Calyc), Campanulaceae (Camp), Goodeniaceae (Good), Menyanthaceae (Menya), Pentaphragmataceae (Penta), Phellinaceae (Phell), Rousseaceae (Rouss), and Stylidiaceae (Styl), respectively. Diagonal cells represented the mean within-family GEDI, whereas off-diagonal cells represented the between-family genetic distances. The color gradient from light yellow to dark blue indicated increasing GEDI values. Gray cells indicated that data for the corresponding gene region were unavailable for the relevant family or family pair, or that the corresponding comparison was not performed.

The family-level GEDI matrices showed that the among-family GEDI of *ITS2*, *trnH-psbA*, and *ycf1* were generally high and usually exceeded their corresponding within-family GEDI; *ITS* also showed pronounced among-family differentiation (Figure 3). In contrast, *rbcL*, *psbA*, and *matR* showed relatively small GEDI differences between among-family and within-family GEDI. These results indicated that highly variable ribosomal spacer regions and some highly variable chloroplast regions contained strong family-level discriminatory signals, whereas relatively conserved CDSs were more likely to maintain within-taxon consistency.

The descriptive diversity statistics were consistent with these patterns. The mean proportion of segregating sites (*Ps*), Watterson’s *Θ*, and nucleotide diversity (*π*) values of the Non-CDSs in Asteraceae were 0.352, 0.0528, and 0.0349, respectively, exceeding the corresponding values of 0.281, 0.0395, and 0.0202 for the CDSs (Table 1). The corresponding values for the Non-CDSs in Asterales were 0.553, 0.0730, and 0.0588, respectively, again exceeding those of 0.448, 0.0635, and 0.0329 for the CDSs (Table 2). The *π* values of *ITS1* were 0.0834 and 0.2214 in Asteraceae and Asterales, respectively, whereas those of *COI* were 0.0041 and 0.0050, respectively.

### 2.2. Candidate Short DNA Barcode Segments and Their Database-Matching Performance

Under the prespecified operational threshold of 0.80, 31 candidate barcode segments (a mean length of 98.55 bp) were retained from the 16 genomic regions (the nucleotide sequences, conservation scores, and screening scores of the barcode segments were provided in Table S1). These candidates represent the output of the specified screening configuration rather than a threshold-independent set. The stability of candidate number, boundaries, and ranking across alternative conservation thresholds was not assessed. The support vector machine (SVM) internal stability scores of all segments exceeded 0.95, and most Mean_cons values ranged from 0.96 to 0.99. Segments from CDSs were generally more conserved; *matR*, *rbcL*, *rpoC1*, and *psbA* retained high identity even under stringent thresholds. Greater variation occurred among Non-CDSs: some segments from *ITS1*, *ITS2*, and *psbK-psbI* combined high conservation, whereas *trnH-psbA* and *trnL-trnF* showed comparatively lower conservation.

**Figure 4:**
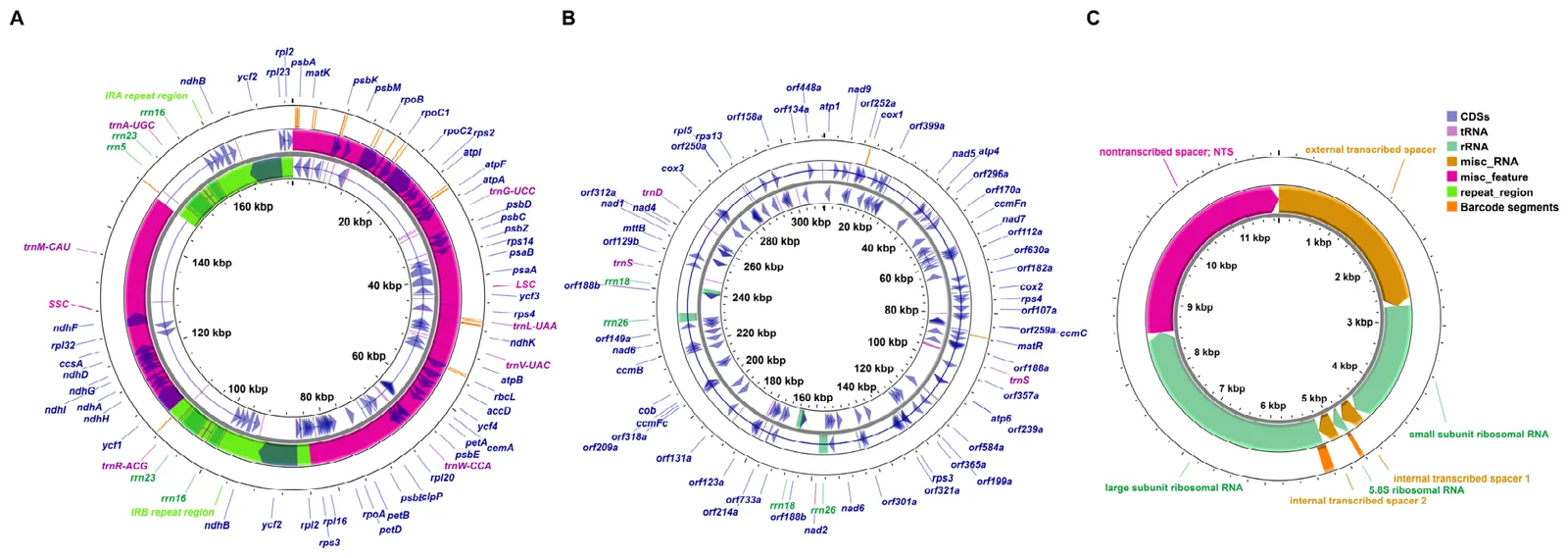
Genomic locations of the barcode segments in the chloroplast genome (**A**), mitochondrial genome (**B**), and nuclear/ribosomal DNA regions (**C**).

**Figure 5:**
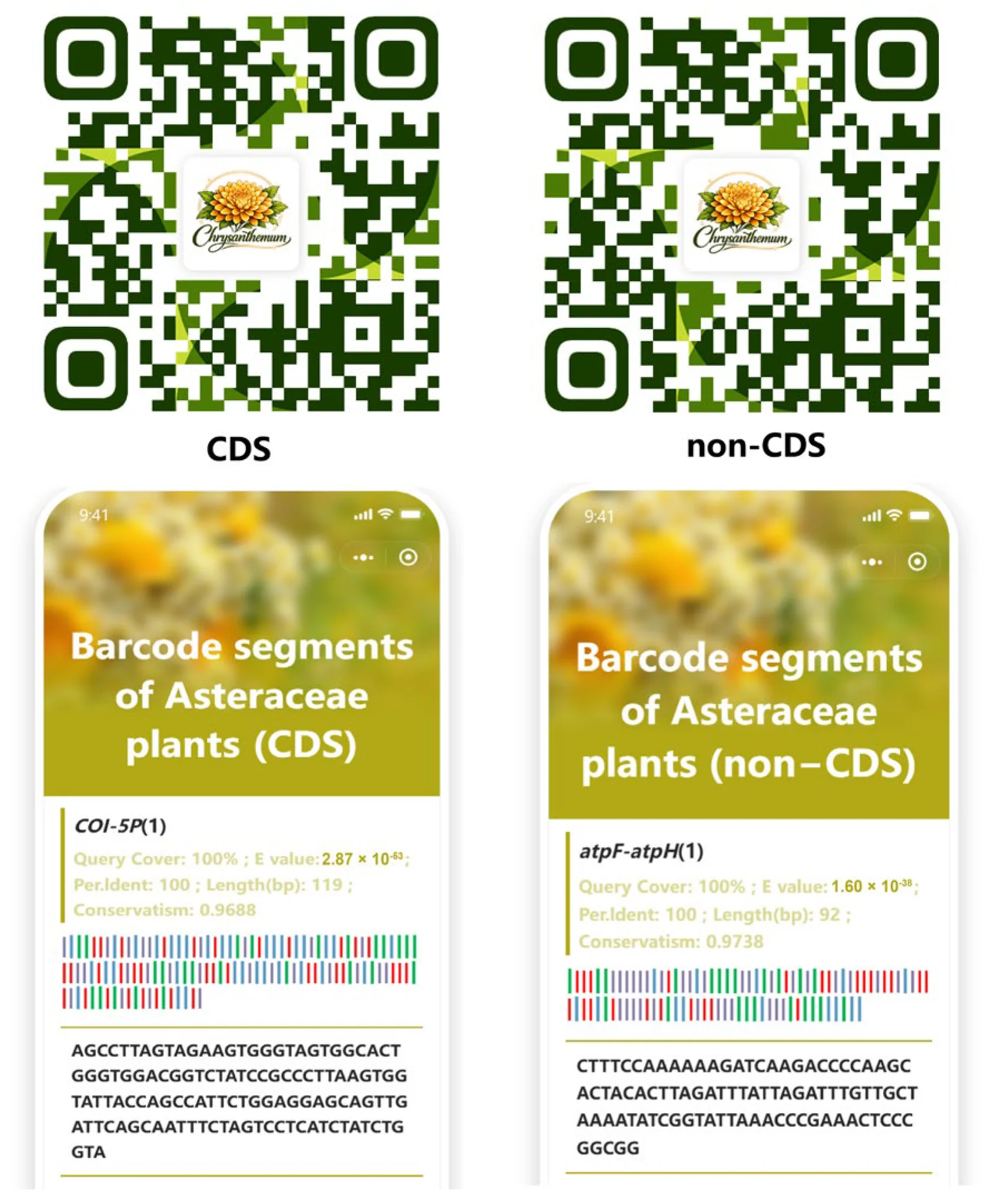
One-dimensional and two-dimensional code representations of the barcode segments.

Under the specified search conditions, the best-match query coverage and sequence identity reached 100% for all segments except *ITS*(1), *trnH-psbA*(1), and *trnL-trnF*(1), and all *E*-values were below 0.001. The reference-sequence locations of the candidate segments were consistent with the expected regions (Figure 4A–C; Table S2). Scanning tests showed that the one-dimensional and two-dimensional barcodes fully presented the segment sequences and their associated information (Figure 5).

### 2.3. Comprehensive In Silico Primer-Amplification Results

All 31 primer pairs generated the expected Asteraceae amplicons in the custom target panel (Table S3). In the examined panels, no primer pair produced a non-Asteraceae Asterales amplicon meeting the predefined high-risk off-target rule (0/31). This panel- and parameter-specific result does not establish universal specificity; per-primer databases, target coverage, mismatches, and all predicted off-target products are reported in Table S3.

For CDS-derived segments, the mean predicted amplicon length was approximately 106 bp. Mean forward- and reverse-primer Tm values were 50.8 and 49.9 °C, and the mean absolute within-pair Tm difference was 3.38 °C. For non-CDS-derived segments, the mean predicted length was approximately 90.7 bp and the mean absolute Tm difference was 7.2 °C. Primer pairs for *atpF*-*atpH*, *psbK*-*psbI*, and *ITS2* showed greater GC-content and Tm variation.

### 2.4. Descriptive Comparison of Barcode-Matching Signals Across Metadata-Defined Metagenomic Sample Groups

Among the 808 metagenomic samples, group A was assigned to the metadata-supported high-confidence target-containing group, group B to the low-abundance or complex-background target-containing group, group C to the non-Asteraceae Asterales background group, and group D to the distantly related background group (Table 3; the basic information and accession number of the metagenomic dataset are detailed in Table S4).

The descriptive matching summaries showed a pronounced gradient among the four groups. Mean query coverage and percent identity were 93.17% and 99.06%, respectively, in group A and 90.86% and 98.41%, respectively, in group B. The corresponding values decreased to 40.45% and 45.59% in group C and to 10.86% and 13.02% in group D. These results indicated stronger barcode-matching signals in the metadata-supported target-containing groups than in the two background groups. Because no independent calibration set, sample-level classification threshold, or final positive/negative decision rule was used, these results represented descriptive between-group separation rather than estimates of diagnostic sensitivity, specificity, or accuracy.

### 2.5. Target-Taxon Recall and Discrimination Against Closely Related Backgrounds

After quality control and cluster-level partitioning, 221,113 sequences were assigned to the discovery dataset and 58,843 to the held-out test dataset. No accession identifier or identical/near-duplicate cluster was shared across sets. Candidate discovery used only the discovery dataset, whereas target-family recall and related-family differentiation used only the held-out test dataset. The accession and cluster audit is provided with the reproducibility files (Table S5).

**Figure 6:**
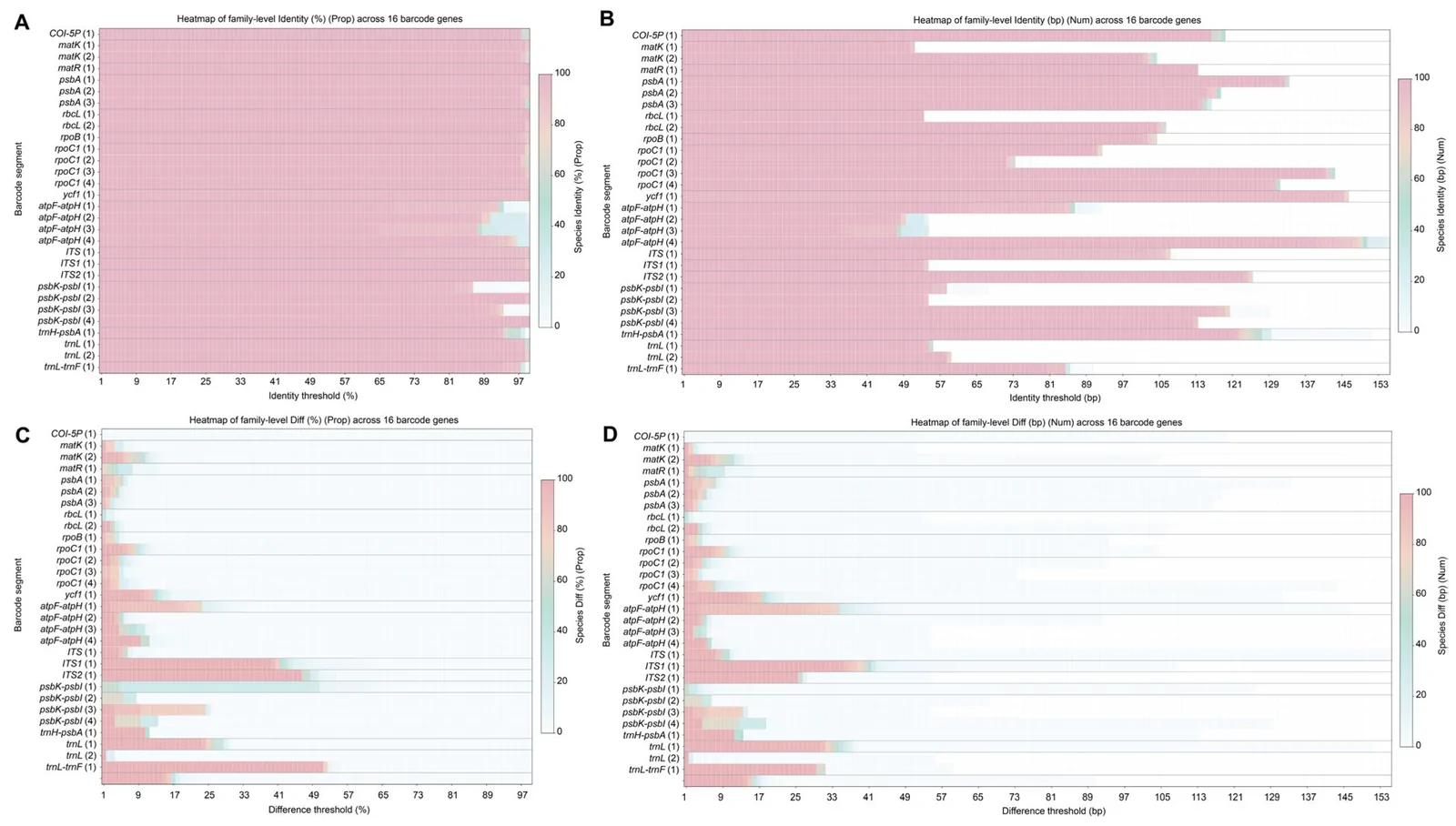
Threshold-dependent within-Asteraceae sequence recall and differentiation from non-Asteraceae Asterales backgrounds. (**A**) Recall at nucleotide-identity percentage thresholds. (**B**) Recall at identical-site-number thresholds. (**C**) Background differentiation at nucleotide-difference percentage thresholds. (**D**) Background differentiation at differing-site-number thresholds. The word ‘Species’ retained in the original software-generated panel headers is a legacy output label and does not indicate species-level identification in this study.

The target-taxon recall analysis showed pronounced differences among segments under stringent identity thresholds (Figure 6). *matR* reached 100.00% for mean nucleotide identity, recall at the >95%, >98%, and >99% identity thresholds, and the proportion of full-length exact matches, thereby showing the highest within-Asteraceae stability (Figure 6A,B). At the >99% identity threshold, the recall rates of *ITS2*, *ycf1*, and *rbcL* were 93.36%, 93.07%, and 92.56%, respectively, and their proportions of full-length exact matches were 78.67%, 77.81%, and 80.82%, respectively. The recall rates of *ITS1*, *rpoB*, *psbA*, *rpoC1*, *matK*, and *trnL* at the >99% identity threshold ranged from 70.44% to 83.23%, and their proportions of full-length exact matches ranged from 46.65% to 82.81%. *atpF-atpH*, *psbK-psbI*, *trnH-psbA*, and *trnL-trnF* showed relatively weak recall, and substantial variation occurred among the different *psbK-psbI* subsegments. *ITS* showed a recall rate of 90.28% at the >99% identity threshold, but its proportion of full-length exact matches was 0, indicating that it was suitable for high-similarity matching but not for use of complete identity as the sole decision criterion.

The analysis of differences from closely related backgrounds showed that the mean nucleotide differences in *ITS1* and *ITS* were 48.12% and 43.33%, respectively, and that 100.00% of sequences reached a difference of at least 20% (Figure 6C,D). The mean nucleotide differences in *trnH-psbA* and *ycf1* were 26.57% and 21.59%, respectively, with corresponding proportions of 96.51% and 80.56%. The mean nucleotide differences in *matR* and *rbcL* were only 4.42% and 2.40%, respectively, and the proportions reaching the 20% difference threshold were both 0.

Joint consideration of within-Asteraceae recall and related-family differentiation produced distinct empirical profiles. *ITS2* and *ycf1* combined relatively high recall with moderate differentiation; *ITS1* showed stronger differentiation but lower exact matching; *matR* and *rbcL* showed high recall but limited differentiation; and *ITS* and *trnH*-*psbA* showed larger background differences. These are descriptive family-level profiles, not predefined screening or confirmatory roles.

### 2.6. Structural Associations Among Conservation, Diversity, and Family Level Differentiation

**Figure 7:**
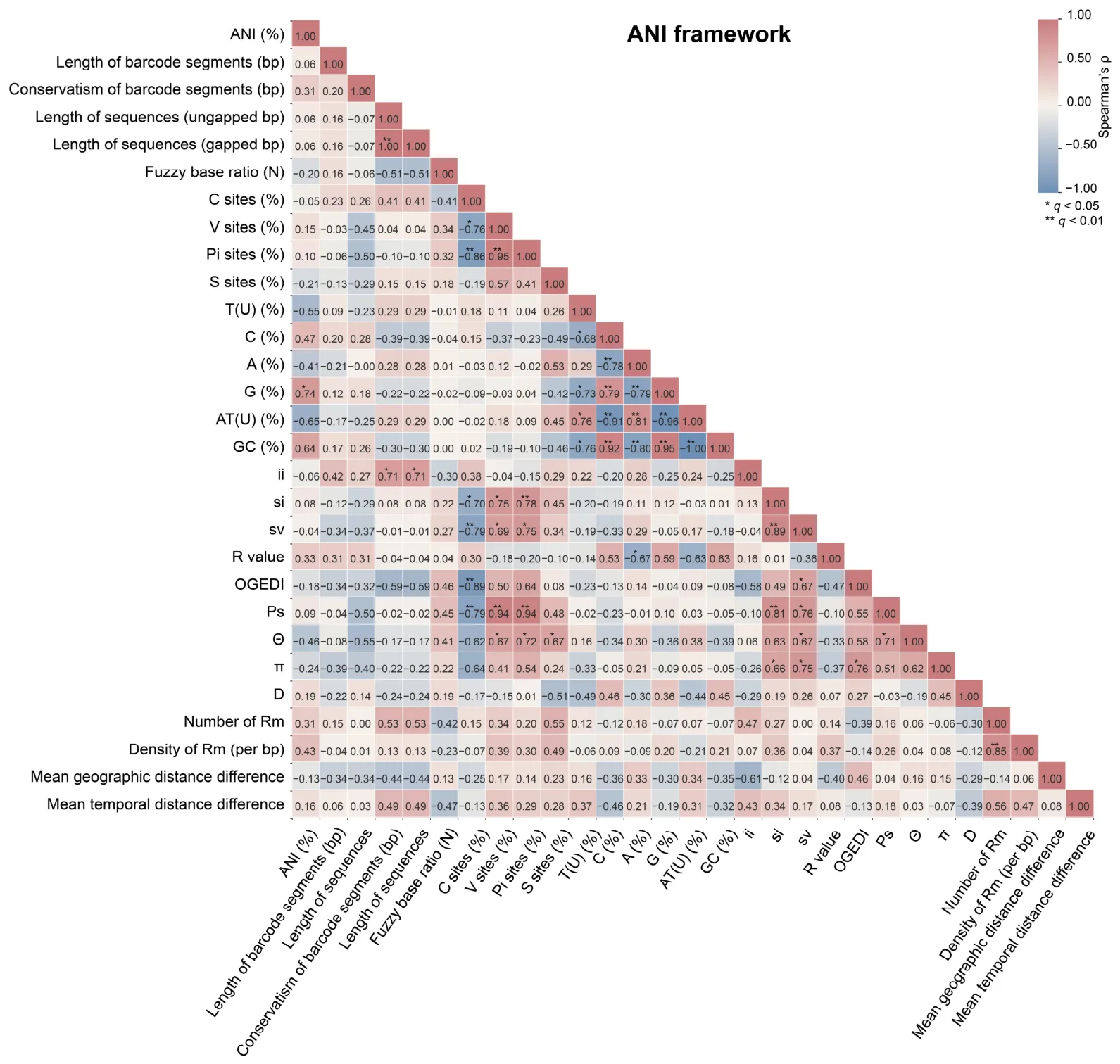
Correlation analysis between genetic characteristics and evolutionary indices of candidate marker regions in Asteraceae under the ANI frameworks. Associations among different genetic and evolutionary indices of candidate marker regions in Asteraceae were evaluated using Spearman’s rank correlation analysis. Colors represented the magnitudes of the correlation coefficients, with red indicating positive correlations and blue indicating negative correlations. After multiple-testing correction using the Benjamini–Hochberg method, significant correlations were marked with asterisks (* *q* < 0.05; ** *q* < 0.01).

**Figure 8:**
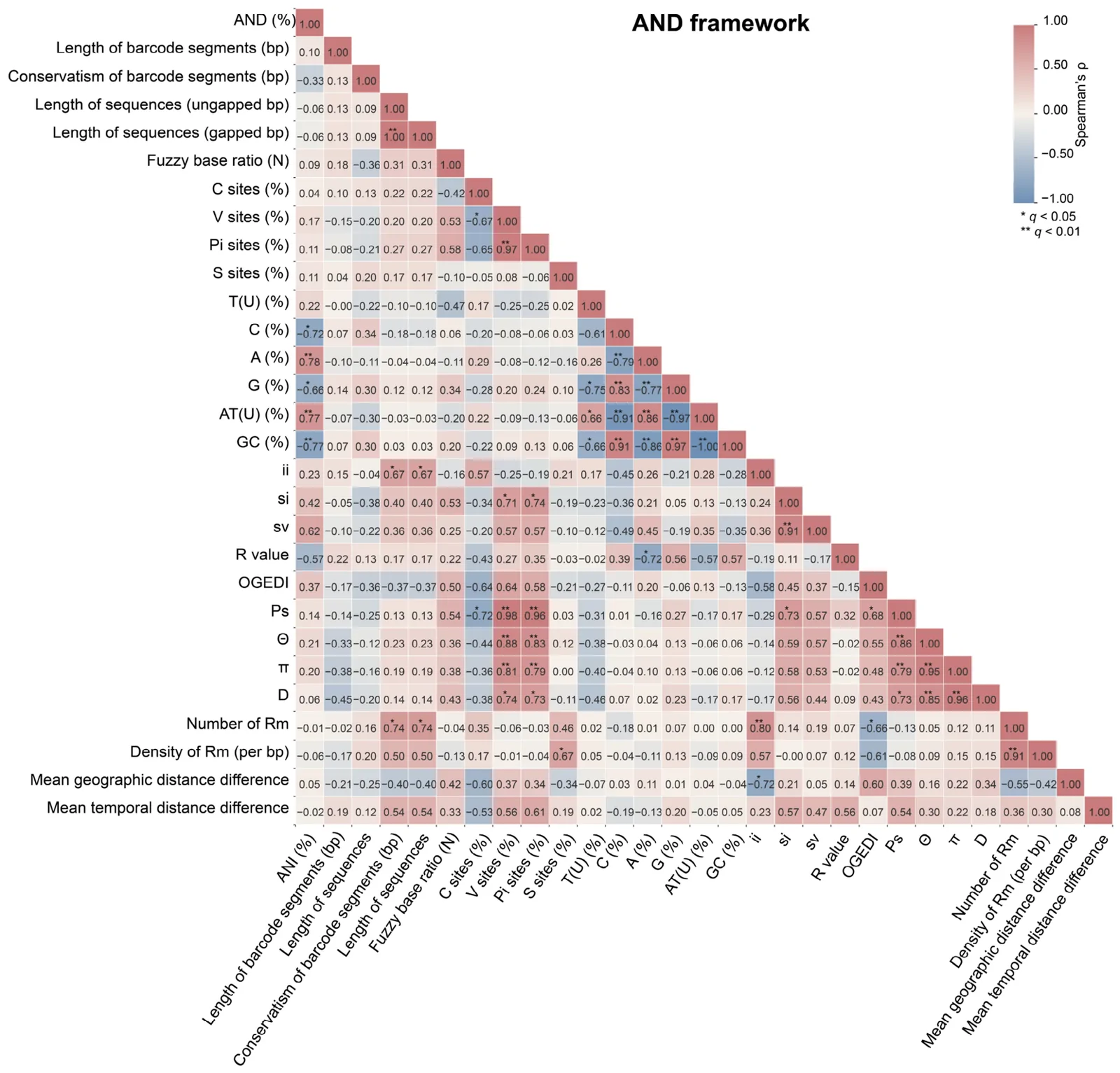
Correlation analysis between genetic characteristics and evolutionary indices of candidate marker regions in Asteraceae under the AND frameworks. Associations among different genetic and evolutionary indices of candidate marker regions in Asteraceae were evaluated using Spearman’s rank correlation analysis. Colors represented the magnitudes of the correlation coefficients, with red indicating positive correlations and blue indicating negative correlations. After multiple-testing correction using the Benjamini–Hochberg method, significant correlations were marked with asterisks (* *q* < 0.05; ** *q* < 0.01).

Under the mean within-taxon nucleotide-identity framework, average nucleotide-level identity (ANI, %) showed a significant positive correlation only with base content. The proportion of conserved sites (C sites) was negatively correlated with among-family GEDI and the proportion of Pi sites, whereas among-family GEDI was positively correlated with *π*, and the proportion of Pi sites was positively correlated with Watterson’s *Θ* (Figure 7). Gapped sequence length was positively correlated with absolute substitution counts, indicating that some count-based metrics were affected by sequence length in cross-region comparisons. Under the mean among-taxon nucleotide-difference framework, average nucleotide-level difference (AND, %) was significantly positively correlated with overall GEDI (OGEDI), whereas *π*, Watterson’s *Θ*, the proportion of variable sites (V sites), and the proportion of Pi sites showed coordinated increases. The minimum recombination events (*Rm*) were also highly correlated with its density (Figure 8). In addition, stratified analysis showed that the negative “conservation–informativeness” association was more stable in CDSs: as the proportion of C sites increased, the proportion of Pi sites, Watterson’s *Θ*, and the degree of among-family differentiation generally decreased. In Non-CDS regions, the correlation structure was less stable because fewer segments were available and the range of variation was greater.

### 2.7. Family Level Phylogenetic Placement Signals of Different Short Segments

**Figure 9:**
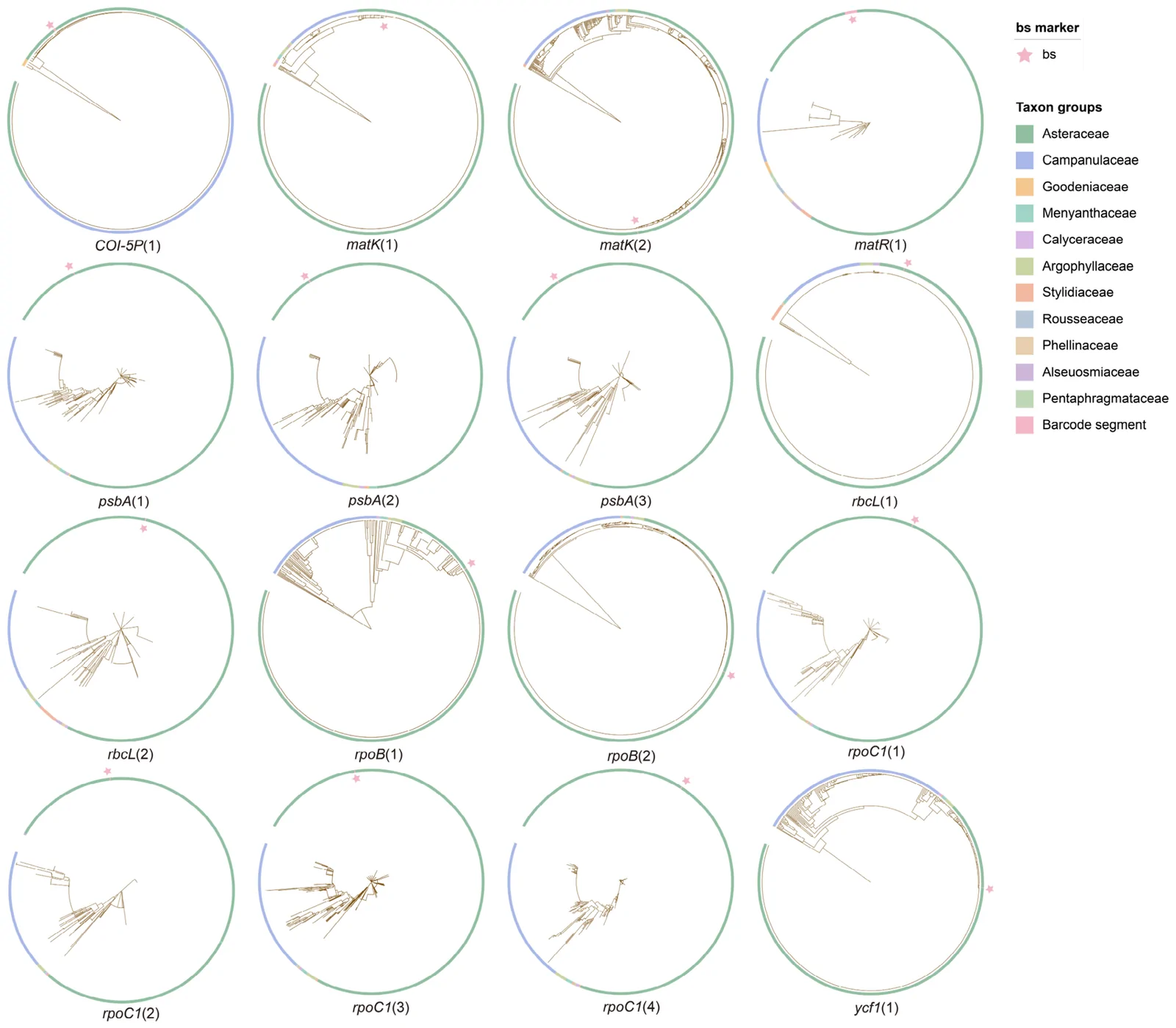
Family level maximum-likelihood placement trees constructed from 8 CDS regions and corresponding candidate short segments across Asterales. These trees were not used to infer species-level identification accuracy.

**Figure 10:**
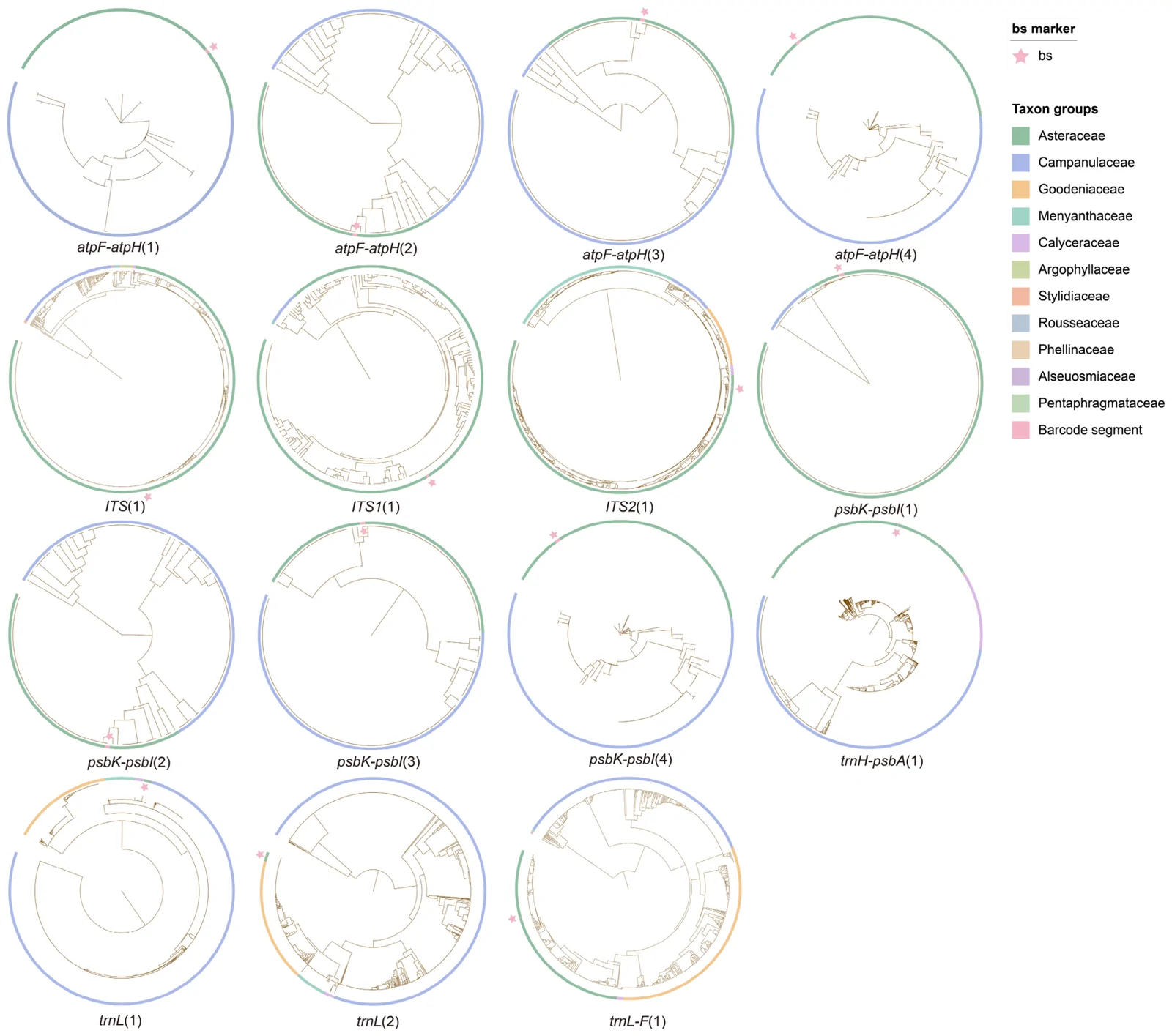
Family level maximum-likelihood placement trees constructed from 8 Non-CDS regions and corresponding candidate short segments across Asterales. These trees were not used to infer species-level identification accuracy.

To evaluate whether the candidate barcode segments retained sufficient phylogenetic information for family-level placement, neighbor-joining (NJ) reference trees were first constructed from the complete sequences of each of the 16 marker regions, excluding the candidate segment queries (Figure S1). Each of the 31 candidate segments was subsequently incorporated into its corresponding complete-region reference dataset, and separate maximum-likelihood (ML) trees were constructed to assess its phylogenetic placement (Figure 9 and Figure 10).

Across the segment-specific ML analyses, all candidate barcode segments were placed within or immediately adjacent to branches containing Asteraceae reference sequences, and none was unambiguously embedded within another major family of Asterales. These results indicated that the short segments generally retained phylogenetic signals supporting their assignment to Asteraceae relative to the sampled non-Asteraceae families of Asterales. However, this placement should be interpreted as evidence of family-level detection rather than species-level discrimination within Asteraceae.

Placement patterns were not fully consistent among candidate segments from the same marker region. Some were nested within Asteraceae-associated branches, whereas others occurred near branch bases or showed weak separation from related families. Segments from *psbA*, *rpoC1*, *matR*, and other conserved regions frequently showed short internal branches, star-like patterns, or weak support. These trees assess family-level placement, not species-level identification or a complete Asterales phylogeny.

## 3. Discussion

This study identified a descriptive gradient between within-Asteraceae sequence stability and differentiation from other Asterales families. Conserved regions more often retained high within-target identity, whereas variable regions more often showed larger differences from related family-level backgrounds. Part of this pattern is mathematically expected because conserved, variable, segregating, and parsimony-informative sites are not independent quantities. The results therefore support comparative profiling of candidate regions but do not establish that evolutionary trade-offs causally shaped barcode performance. The study integrated short-segment screening, family-level target recall, exclusion of non-Asteraceae Asterales backgrounds, exploratory metagenomic matching, and family-level phylogenetic placement. Its contribution is a multidimensional comparison rather than a claim that one locus is universally best. Because marker roles were assigned after observing the same performance profiles, the data do not yet justify an operational hierarchy of screening and confirmatory markers.

This study extends earlier comparisons by integrating short-segment screening, within-Asteraceae sequence recall, exclusion of non-Asteraceae Asterales backgrounds, exploratory metagenomic matching, and family-level phylogenetic placement [17,20]. The resulting profiles can guide hypotheses for later assay development, but they do not establish operational screening or confirmatory roles because marker categories and application weights were not predefined.

The differences among *ITS1*, *ITS2*, and *ycf1* illustrate the observed profiles. *ITS1* showed the largest mean difference from non-Asteraceae Asterales backgrounds but lower exact matching than *ITS2*. *ITS2* and *ycf1* combined relatively high within-Asteraceae recall with moderate background differentiation. These statements are restricted to the family-level comparison used here. In addition, full *ITS*, *ITS1*, and *ITS2* overlap physically and are not independent loci; retaining all three as alternative candidate definitions increases representation of the ribosomal cistron and requires sensitivity analysis [7,8,11].

High within-Asteraceae identity in *matR* and *rbcL* identifies them as candidates for broad target recovery, but the present data do not support stand-alone confirmatory use. Formal marker-role assignment should use a prespecified loss function or Pareto-front analysis in discovery data and independent testing. Decision-curve analysis would become appropriate only after a validated binary endpoint and operational prediction model are available [20,21].

The public metagenomic comparison provided an external-context plausibility check rather than diagnostic validation. Public metadata did not independently establish the presence or absence of Asteraceae DNA [16,17,18,19,22]. A latent-class model was not fitted because the dataset lacked multiple conditionally independent assays required for reliable identification [23]. Future validation should use voucher-linked morphology, whole-genome, plastome evidence, spike-ins, or orthogonal assays.

The current ranking is post hoc. Before assigning operational screening or confirmatory categories, future studies should define objective weights, calculate a Pareto frontier using held-out recall, nontarget exclusion, primer feasibility, and uncertainty, and report bootstrap stability [23,24].

The phylogenetic comparisons were not designed to infer the complete evolutionary history of Asterales. Mechanistic explanations would require locus-appropriate analyses. For CDSs, this could include orthology-checked, codon-aware dN/dS models stratified by branch or site; for Non-CDSs, conserved-element annotation and substitution-rate models would be more appropriate. Recombination summaries calculated across mixed species are descriptive and cannot be interpreted as population recombination rates. These analyses are beyond the current descriptive scope [25,26].

From an applied perspective, the screened segments should be regarded as candidates for assay development. Any future use in medicinal-plant identification, food traceability, or environmental DNA monitoring requires comprehensive primer-specificity assessment and experimental testing of amplification efficiency, limit of detection, repeatability, mixture performance, and cross-region robustness [27].

**Figure 11:**
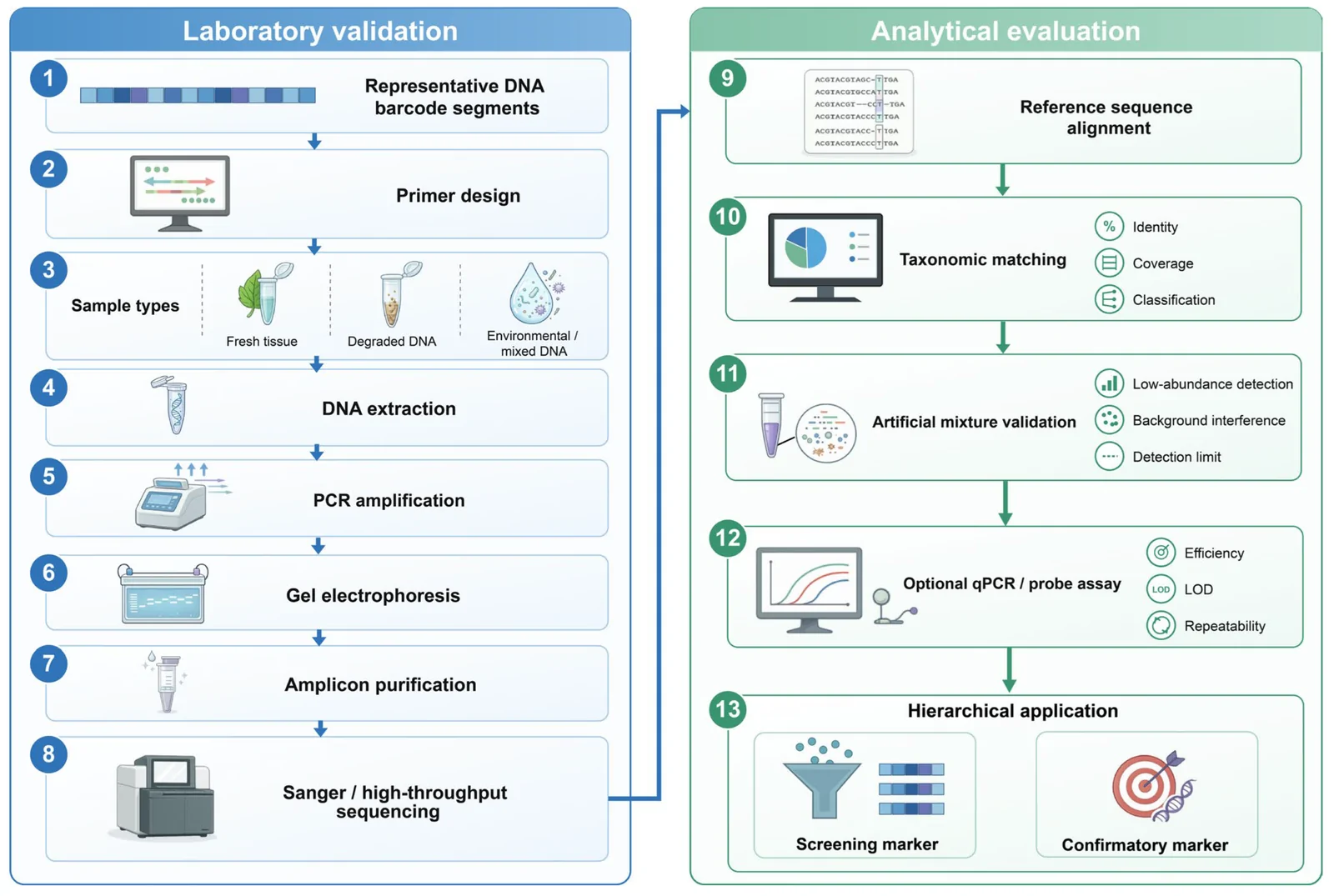
Proposed workflow for future experimental validation of candidate short DNA barcode segments; no experiments in this workflow were performed in the present study.

The following workflow is proposed for future experimental validation and was not performed in the present study (Figure 11). Representative candidate segments should first be selected using prespecified criteria and an independent computational test set. Primer pairs should then undergo comprehensive genomic off-target assessment before laboratory testing [3,4,16]. Voucher-linked Asteraceae tissues, closely related non-Asteraceae Asterales, degraded templates, and complex environmental samples should be tested. PCR products should be evaluated by gel electrophoresis and confirmed by Sanger or amplicon sequencing. Taxonomically dense congeneric sampling is required before any species-level claim can be made [5,6,10,15].

Artificial mixtures and dilution series should quantify the limit of detection, background interference, replicate consistency, and false-positive amplification. Morphological vouchers, whole-genome or plastome evidence, or orthogonal assays should establish target presence and absence independently of the candidate barcodes [17,18,19]. Candidate segments may subsequently be developed for qPCR or probe-based detection, with amplification efficiency, limit of detection, limit of quantification, and repeatability reported according to applicable assay guidelines [22,27]. Operational screening and confirmation roles should be assigned only after prospective validation and prespecified decision analysis.

A principal limitation of this study was that the performance of the candidate barcode segments was evaluated mainly using public sequence databases and computational analyses; therefore, their amplification efficiency, analytical sensitivity, specificity, and ecological applicability still require validation across a broader range of real-sample scenarios. In addition, candidate-segment discovery relied on a single operational conservation threshold of 0.80, corresponding to normalized Shannon entropy ≤ 0.20. Although this threshold was applied uniformly to restrict the screening to relatively low-entropy alignment positions, it was not a biologically validated or universally optimal cutoff. Because alternative threshold settings were not evaluated, the number, coordinates, length distribution, and performance ranking of the 31 candidate segments should be interpreted as conditional on the specified screening configuration rather than as threshold-independent or universally robust results. Moreover, although a systematic multi-metric evaluation framework was established, its generalizability requires further assessment using independent, accession-separated external datasets and cross-regional validation. Future studies should therefore systematically evaluate alternative conservation thresholds, repeat both candidate discovery and downstream performance assessment using independent datasets, and validate representative segments experimentally. Combining such validation with continuously updated sequence resources and data-driven models could ultimately enable more precise, robust, and dynamically optimized barcode-design strategies [20,22].

## 4. Materials and Methods

### 4.1. Overview of the Study Design and Workflow

**Figure 12:**
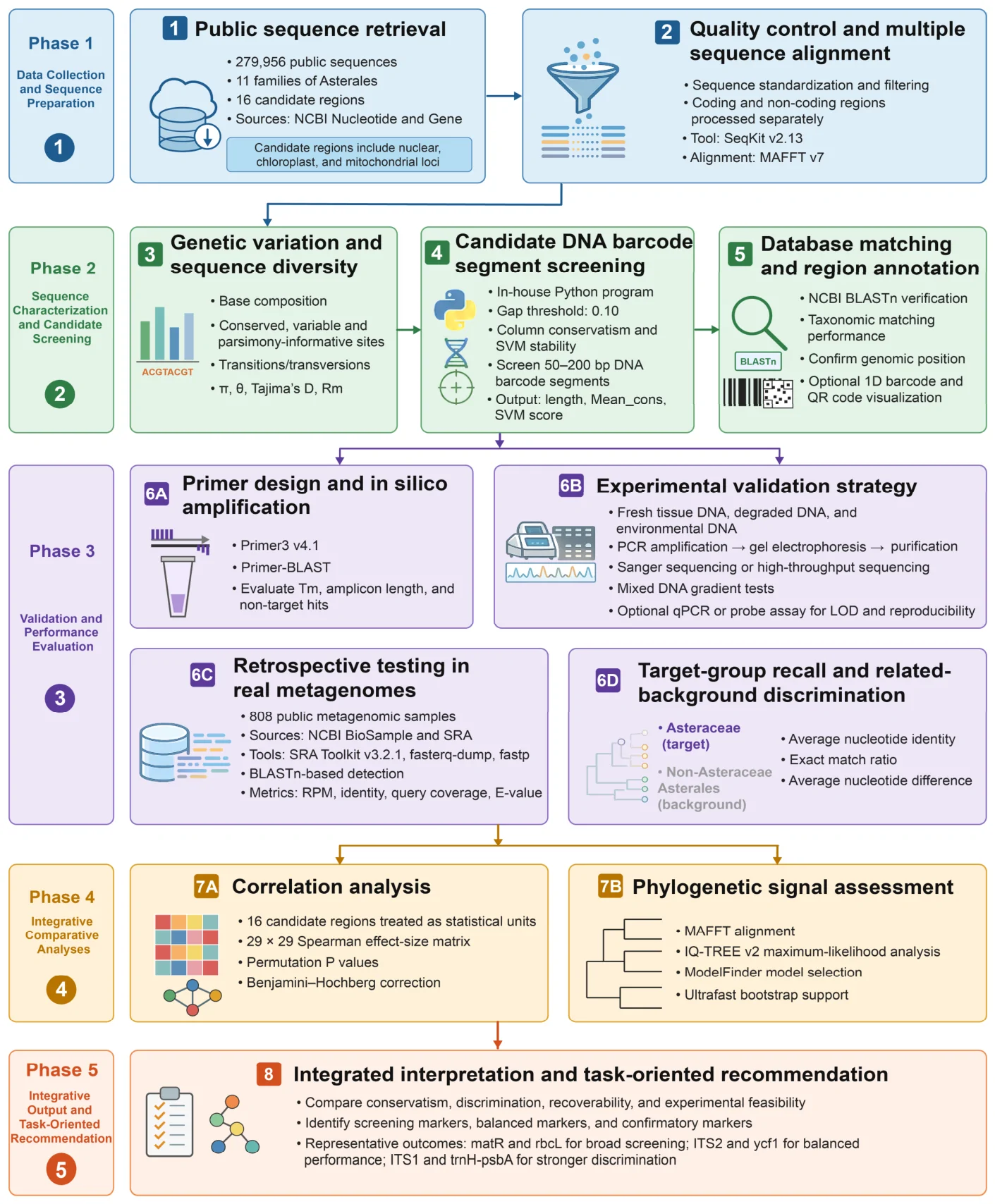
Workflow for the screening and multidimensional evaluation of DNA barcode segments in Asteraceae plants.

A systematic framework was used to compare candidate short DNA barcode segments for Asteraceae detection within Asterales (Figure 12). Public sequences were quality controlled and aligned; segments were screened using conservation and length criteria; and descriptive variation, internal matching, within-Asteraceae recall, non-Asteraceae Asterales exclusion, exploratory metagenomic matching, and family-level phylogenetic placement were compared. The workflow did not evaluate species identification within Asteraceae.

### 4.2. Sequence Retrieval, Quality Control, and Multiple-Sequence Alignment

Sixteen candidate DNA barcode regions were retrieved for plants from 11 families of Asterales (Alseuosmiaceae, Argophyllaceae, Asteraceae, Calyceraceae, Campanulaceae, Goodeniaceae, Menyanthaceae, Pentaphragmataceae, Phellinaceae, Rousseaceae, and Stylidiaceae) from the Nucleotide and Gene databases of the National Center for Biotechnology Information (NCBI, https://www.ncbi.nlm.nih.gov/, accessed on 10 January 2026) [28,29]. The CDSs included *COI*/*COI-5P*, *matK*, *matR*, *psbA*, *rbcL*, *rpoB*, *rpoC1*, and *ycf1*, whereas the noncoding sequences (Non-CDSs) included *atpF-atpH*, *ITS*, *ITS1*, *ITS2*, *psbK-psbI*, *trnH-psbA*, *trnL*, and *trnL-trnF*. As of 20 January 2026, the 176 NCBI Nucleotide queries yielded 279,956 locus–record matches. After deduplication by NCBI UID across queries and loci, 135,942 unique sequence records remained. Taxonomic validation retained 134,372 records assigned to the intended 11 families of Asterales. These records represented 14,886 unique named species-level taxa belonging to 1706 genera, including 14,745 non-hybrid species and 141 named hybrid taxa. An additional 3442 records lacked an unambiguous species-level assignment and were therefore excluded from the species-level taxon counts. Exact queries, dates, accession lists, raw and deduplicated records, quality-controlled sequences, species-level taxa, genera, hybrids, and ambiguous records for every locus were reported in Table S5.

After sequence retrieval, standardized quality control was performed using SeqKit v2.13 [30]. For CDSs, clearly incomplete or abnormally extended sequences were removed. For *ITS* and intergenic spacer regions, sequence annotations were used to determine whether abnormal lengths resulted from genuine insertion–deletion variation, and only incomplete, misannotated, or clearly anomalous records were removed. Multiple-sequence alignments were then performed separately for each candidate region using MAFFT v7 [31] with default parameters.

Before candidate-segment discovery, the quality-controlled sequences for each locus were divided into nonoverlapping discovery and test datasets. The discovery dataset was used exclusively for multiple-sequence alignment, site-conservation estimation, SVM-assisted screening, and determination of candidate-segment coordinates. The test dataset was withheld from all candidate-discovery procedures and was used only for the subsequent evaluation of target-group recall and differentiation from non-Asteraceae Asterales backgrounds.

To prevent information leakage, identical and near-duplicate sequences were identified across the combined dataset using SPS v1.0 software [27] (https://github.com/JS-HNU/SPS, accessed on 10 January 2026) with thresholds of ≥80% sequence identity and ≥80% alignment coverage (Refer to the research conducted by Jiang et al.) [17,21,27]. All sequences belonging to the same duplicate or near-duplicate cluster were assigned entirely to either the discovery dataset or the test dataset. Dataset assignments were performed at the cluster level rather than at the individual-record level. Accession numbers and cluster identifiers were subsequently cross-checked, confirming that no accession or near-duplicate sequence cluster occurred in both datasets.

### 4.3. Characterization of Genetic Variation and Sequence Diversity

Descriptive analyses of genetic variation were performed for the candidate regions at the Asteraceae and Asterales levels using MEGA v12 and DnaSP v5 [32,33], respectively. The statistical metrics included sequence length, nucleotide composition, C sites, V sites, Pi sites, segregating sites (S sites), the number of identical pairs (II), transitionsal pairs (SI) and transversional pairs (SV), nucleotide diversity (*π*), *Ps*, Watterson’s *Θ*, Tajima’s *D*, and the minimum number of *Rm* [34,35,36]. Because the data spanned multiple species and taxonomic units, Tajima’s *D* and *Rm* were used only as descriptive indicators of sequence-variation structure and were not used to directly infer population-level selection or actual recombination processes. To compare within-family and among-family differentiation across candidate regions, family-level GEDI and OGEDI matrices were calculated [37].

For locus *l*, let d_l_(*x*_i_,*x_j_*) be the pairwise nucleotide distance after alignment filtering, and let *n_a_* and *n_β_* be retained sequence counts for families a and b.

$${GEDI}_{l}(a,a)=\frac{2}{n_{a}(n_{a}-1)}\underset{1\leq i<j\leq n_{a}}{\sum }d_{l}(x_{i},x_{j})$$

$${GEDI}_{l}(a,b)=\frac{1}{n_{a}n_{b}}\sum_{i=1}^{n_{a}}\sum_{j=1}^{n_{b}}d_{l}(x_{i},x_{j}),a\neq b$$

$${OGEDI}_{l}=\frac{1}{\left|P_{l}\right|}\underset{(a,b)\in P_{l}}{\sum }{GEDI}_{l}(a,b)$$

The first expression is the mean within-family distance; the second is the mean interfamily distance. *P_l_* is the set of unordered family pairs with valid data; OGEDI_l_ is their unweighted mean. (The detailed calculation formulas for GEDI and OGEDI can be found in File S1).

Sample sizes were reported according to the observational unit used in GEDI analysis. The numbers of sequences, valid sequence pairs, and represented family pairs contributing to each GEDI and OGEDI estimate were reported in Table S5. Uncertainty in GEDI and OGEDI was quantified using 2000 stratified bootstrap replicates. Sequences were resampled with replacement within each family, and all corresponding genetic-distance indices were recalculated for each replicate. The 2.5th and 97.5th percentiles of the bootstrap distributions were used as the 95% confidence limits.

### 4.4. Screening of Candidate Short Barcode Segments and Database Matching

All candidate barcode segments were identified exclusively from the discovery dataset. No sequence assigned to the held-out test dataset contributed to the estimation of alignment-column entropy, SVM model fitting, candidate-segment ranking, or determination of segment boundaries. Candidate short barcode segments were screened using an in-house program developed in the laboratory with Python and scikit-learn (Barcoding v1.0, https://github.com/Tetingp/Barcoding1/releases/tag/Barcoding1, accessed on 25 January 2026) [21,38].

The gap threshold for columns in the multiple-sequence alignments was set to 0.10; positions with gap proportions greater than 10% were removed. This ensured that positions included in the screening had sequence coverage of at least 90% and reduced the effects of insertion-deletion-rich regions and unstable alignment positions on conservation estimates. For the small number of gaps retained at individual positions, the program determined a consensus base from the frequencies of non-gap nucleotides in that column and used it for imputation; thus, this threshold also limited the proportion of imputed data at any individual position to less than 10%. Conservation at each alignment position was calculated from normalized Shannon entropy [37], with site conservation defined as 1 minus the normalized entropy. The conservation threshold was set to 0.80, meaning that only low-entropy positions with normalized entropy no greater than 0.20 were retained. This parameter was not equivalent to 80% nucleotide identity but instead reflected the concentration of nucleotide composition at a position. The threshold was intended to exclude highly variable positions while avoiding conditions so stringent that continuous candidate regions of sufficient length could not be formed. Candidate segment lengths were restricted to 50–200 bp. This range jointly considered the greater amplifiability and recoverability of short segments from degraded DNA and environmental samples and the possibility that excessively short sequences would lack sufficient taxonomic information [21]. These parameters served as uniform operational screening criteria to balance alignment reliability, target-taxon coverage, segment amplifiability, and taxonomic resolution; they did not represent universally optimal parameters for all gene regions or taxonomic levels. In addition, the conservation threshold was intended to restrict candidate discovery to relatively low-entropy alignment positions, but it was not derived as a biologically validated or universally optimal cutoff. Therefore, the number, coordinates, and composition of the resulting candidate segments should be interpreted as conditional on this parameter setting. Alternative conservation thresholds were not evaluated in the present study.

The output also included segment length, mean conservation (Mean_cons), and the SVM internal stability score, which were used for preliminary ranking of candidate segments. Each alignment column was represented by four nucleotide-composition features: GC proportion, purine proportion, maximum nucleotide frequency, and the number of observed nucleotide states. Pseudo-labels were assigned according to conservation scores calculated from column-level Shannon entropy: columns with scores at least 0.02 above the predefined threshold were labeled highly conserved, whereas columns with scores at least 0.02 below the threshold were labeled nonconserved; columns with intermediate scores were excluded from model training. An RBF-kernel support vector machine with class-balanced weights was evaluated using stratified cross-validation of up to five folds [21,38], with the mean cross-validated ROC-AUC used as the internal evaluation metric. The final model was fitted to all labeled columns and used to estimate the probability of high conservation for each alignment column. The mean probability within each candidate region was reported as an SVM-assisted consistency score.

The screened segments were submitted to NCBI BLASTn web server [39] (last updated: February 2026; accessed on: 10 February 2026; core data: Core nucleotide database; default parameters), and their taxonomic matching performance was evaluated against the Standard Nucleotide database. The genomic positions of the candidate segments were also verified according to reference-sequence annotations. To facilitate presentation and information linkage, the candidate segments were converted into one-dimensional nucleotide graphics and two-dimensional quick-response codes. These visualizations were used only for information display and were not treated as metrics for evaluating candidate-segment performance.

### 4.5. Primer Design and Comprehensive In Silico Amplification Evaluation

Forward and reverse primers for all 31 candidate barcode segments were designed using Primer3 v4.1 [40], with melting temperatures (*Tm*) constrained to 40–60 °C. The specificity of each predesigned primer pair was subsequently evaluated using NCBI Primer-BLAST [41,42,43] against three complementary sequence panels. First, target amplification and discrimination from closely related taxa were assessed using a custom database containing sequences from Asteraceae and the other 10 Asterales families included in this study: Alseuosmiaceae, Argophyllaceae, Calyceraceae, Campanulaceae, Goodeniaceae, Menyanthaceae, Pentaphragmataceae, Phellinaceae, Rousseaceae, and Stylidiaceae. Nuclear genomic and nucleotide records were used for *ITS*, *ITS1*, and *ITS2*; complete mitochondrial genomes were used for *COI-5P* and *matR*; and complete plastid genomes were used for the remaining plastid coding and noncoding regions. The use of complete organellar genomes ensured that intergenic regions, including *atpF*-*atpH*, *psbK*-*psbI*, *trnH*-*psbA*, and *trnL*-*trnF*, were included even when they were not explicitly annotated in the sequence titles.

Second, potential off-target amplification across plants was examined using the NCBI “Genomes for selected eukaryotic organisms (primary assemblies only)” database restricted to Viridiplantae (taxid: 33090), which included available nuclear, mitochondrial, and plastid genomic sequences. Third, an unrestricted core_nt search was performed to identify additional plant and non-plant off-targets; uncultured and environmental sequences were not excluded. The minimum number of mismatches required for an unintended target was set to two, the maximum target amplicon size was set to 1000 bp, and all other Primer-BLAST parameters were retained at their default settings. Searches were completed on 25 August 2026.

A predicted amplicon was recorded only when the forward and reverse primers matched the same subject sequence in opposite, inward-facing orientations and generated a product of 50–1000 bp. A potential high-risk off-target was defined as a non-Asteraceae template for which both primers contained no more than two mismatches and showed complete identity within the terminal five nucleotides at their 3′ ends. For each primer pair, primer length, Tm, GC content, the Tm difference between the forward and reverse primers, predicted amplicon length, target coverage, the number and taxonomic distribution of off-target products, and primer–template mismatches were recorded. Sequence-level and species-level results were summarized separately to minimize bias caused by uneven representation and duplicate records in public databases.

### 4.6. Exploratory Evaluation of Barcode-Matching Signals in Public Metagenomic Samples

Publicly available metagenomic samples related to Asterales or suitable as background controls were retrieved from NCBI BioSample (https://www.ncbi.nlm.nih.gov/biosample, accessed on 3 February 2026) and the Sequence Read Archive (SRA) [44,45]. On the basis of the information provided in the BioSample and SRA metadata, the 808 samples with priority given to relatively high-quality samples that complied with metadata standards such as MIMAG, MIGS, MIMARKS, and MIMS were assigned a priori to four descriptive groups: metadata-supported high-confidence target-containing samples (group A), metadata-supported low-abundance or complex-background target-containing samples (group B), non-Asteraceae Asterales background samples (group C), and distantly related background samples (group D) [46,47]. These group labels reflected the expected sample composition reported in the public metadata and were not treated as independently verified diagnostic ground truth. No samples were reserved as an independent negative calibration set. Sampling locations covered multiple countries, principally China, the United States, Japan, and Spain; collection years ranged from 1992 to 2024; and sample sources included plant tissues, plant-associated growth environments, microbial communities, and distantly related ecological backgrounds. Across all samples, Total Spots, Total Bases, mean read length (AvgLength), and data volume (Size_MB) were 11,524,927,746, 2.94719 × 10^12^, 526.13, and 1175,195, respectively. Details of sample classification and the search strategy were provided in Table S4.

Raw data were downloaded using prefetch in SRA Toolkit v3.2.1 [45,48] and converted to FASTQ format using fasterq-dump v3.4.1. Quality control was then performed using fastp v1.3 [49], which automatically identified and removed paired-end sequencing adapters and applied terminal quality trimming with a 4 bp sliding window; reads were truncated when the mean Phred quality score within the window fell below 20. A read was removed when more than 30% of its bases had quality scores below Q20, when it contained more than five N bases, or when its post-quality-control length was less than 50 bp. A local nucleotide BLAST database was then constructed separately for each quality-controlled metagenomic sample, and the candidate short barcode sequences were used as queries. Local nucleotide databases were built for each sample using makeblastdb v2.2.25 from BLAST+ [39]. DUST low-complexity filtering was disabled, and only the highest-scoring segment pair was retained for each query–subject combination. Output fields included query and subject identifiers, sequence identity, alignment length, number of mismatches, number of gaps, alignment coordinates, *E*-value, bit score, query length, and query coverage. These BLAST parameters were used as search and highest-scoring segment pair-retention criteria rather than as thresholds for classifying entire samples as positive or negative.

For multiple highest-scoring segment pairs generated between the same read and the same barcode segment, only the match with the highest bit score was retained. When bit scores were equal, the best match was selected sequentially according to percent identity, query coverage, *E*-value, and alignment length. The resulting matching signals were summarized descriptively within the four metadata-defined sample groups. No barcode-specific background threshold, reads per million-based decision rule, multilocus sample-level classifier, or indeterminate category was applied in the present analysis. Consequently, no samples were excluded as indeterminate, and no confusion matrix or diagnostic-accuracy statistics were calculated. Accordingly, the number of calibration samples and indeterminate cases was not applicable to the analysis actually performed.

### 4.7. Target-Taxon Recall and Related-Family Background Differentiation

To evaluate the sequence stability of candidate short barcode segments within Asteraceae and their ability to distinguish other families of Asterales, an Asteraceae target-sequence set and a closely related background-sequence set comprising non-Asteraceae Asterales were constructed separately. Performance evaluation was conducted using only the held-out test dataset. Candidate-segment coordinates derived from the discovery dataset were mapped to homologous positions in the test sequences, after which target-group recall and related-background differentiation metrics were calculated without modifying the candidate definitions or screening parameters. Each candidate barcode segment was merged with the corresponding target or nontarget reference sequences, and multiple-sequence alignment was performed using MAFFT v7 [31]. According to the start and end coordinates of the candidate barcode in the alignment, all reference sequences were vertically trimmed to obtain homologous regions corresponding to the candidate-segment coordinates and having the same length. The candidate-barcode sequence was used only to assist in determining alignment coordinates and was removed from the reference-sequence set after trimming to prevent self-matching from biasing the identity results.

Recall in the target-sequence set was described using mean nucleotide identity, recall proportions at different identity thresholds, and the proportion of full-length exact matches. Discrimination against closely related backgrounds (the non-Asteraceae closely related background-sequence set) was described using mean nucleotide difference and the proportion of sequences reaching predefined difference thresholds. A position was defined as valid for comparison only when both the candidate segment and the reference sequence contained A, C, G, or T at the same position. Positions containing gaps, N, or other ambiguous bases were excluded from calculations of match and mismatch proportions. Reference sequences shorter than the candidate segment were excluded, whereas longer input sequences were truncated according to the candidate-segment length; thus, all sequences entering this module had been pre-trimmed to homologous coordinates. Detailed algorithms for identity and difference were provided in File S1, and the Python scripts used were publicly available in the GitHub repository (Barcoding v1.0, https://github.com/Tetingp/Barcoding1/releases/tag/Barcoding1, accessed on 25 January 2026) [21,38].

### 4.8. Correlation and Structural-Association Analysis

To characterize associations among within-taxon conservation, sequence composition, genetic diversity, taxonomic differentiation, recombination signals, and the spatiotemporal structure of sampling for the candidate short DNA barcode segments, the 16 candidate genes or spacer regions were treated as independent statistical units. Full *ITS*, *ITS1*, and *ITS2* are overlapping representations of one ribosomal cistron and were not considered independent biological loci. Correlations involving definitionally linked, compositional, or formula-derived variables were labeled structural and were not used to infer evolutionary mechanisms. When multiple short segments were screened from the same candidate region, ANI, AND, Length of barcode segments, and Conservatism of barcode segments were first calculated at the segment level and then averaged to the candidate-region level. All other metrics were derived from the corresponding complete candidate regions, thereby preventing multiple short segments from the same region from being repeatedly weighted.

The correlation analysis included two alternative core metrics, ANI and AND, together with 28 auxiliary metrics: Length of barcode segments (bp), Conservatism of barcode segments (bp), Length of sequences (ungapped bp), Length of sequences (gapped bp), Fuzzy base ratio (N), C sites (%), V sites (%), Pi sites (%), S sites (%), T(U) (%), C (%), A (%), G (%), AT(U) (%), GC (%), II, SI, SV, *R* value, OGEDI, *Ps*, *Θ*, *π*, *D*, Number of *Rm*, Density of *Rm* (per bp), the mean difference in the overall geographic-distance matrix among sampled records, and the mean difference in the overall temporal-distance matrix among sampled records. To avoid deterministic collinearity caused by homologous calculations and oppositely directed relationships, ANI and AND were not entered simultaneously into the same inferential matrix. Instead, separate ANI and AND frameworks were constructed; each framework contained one core metric and 28 auxiliary metrics, for a total of 29 variables. The algorithms for the mean difference in the overall geographic-distance matrix and the mean difference in the overall temporal-distance matrix among sampled records were provided in File S1.

Complete results were reported as matrices rather than only as significant associations. Infinite values, undefined statistics, and results generated by division by zero were uniformly recorded as missing values (NA), and no imputation using the mean, median, or any other method was performed. Each analytical set produced a 29 × 29 Spearman ρ effect-size matrix, the corresponding permutation *p*-value matrix, a Benjamini–Hochberg-adjusted [50] *q*-value matrix, and a matrix of pairwise valid sample sizes (*n_ij_*); a Pearson *r* matrix was reported as a supplementary robustness result. For variable pairs with overlapping definitions, compositional closure, or formula-derived relationships—including ANI and AND; the A/T(U)/C/G compositional proportions; AT(U) and GC; C sites and V sites; Pi sites and S sites; Number of *Rm* and Density of *Rm*; and gapped length, ungapped length, and Fuzzy base ratio—the correlations were interpreted only as structural associations and were not used to propose independent evolutionary mechanisms. These analyses were performed in R using the Hmisc v5.2, Coin v3, and Corrplot v0.9 packages.

For each ANI and AND framework, the observational unit was the marker region (overall *n* = 16; CDS subset *n* = 8; non-CDS subset *n* = 8). Missing or undefined values were excluded pairwise and were not imputed. Spearman’s rank correlation coefficient (rho) was the primary statistic. For the full dataset, *p* values were obtained using the standard Spearman test; for the *n* = 8 subsets, exact *p* values were obtained by exhaustive enumeration of 40,320 permutations. *p* values were adjusted using the Benjamini–Hochberg procedure, and Pearson’s r and its *p* value were reported as a sensitivity analysis. Complete pairwise coefficients and inferential results are provided in Table S6.

### 4.9. Assessment of Family Level Phylogenetic Placement

The candidate short barcode segments and their corresponding full-length gene regions were separately aligned using MAFFT v7 [31], and the same taxa were retained in the short-segment and full-region datasets. The primary analysis used a partial-deletion strategy in which alignment positions with more than 5% gaps or unknown bases were removed; a complete-deletion strategy was also used for sensitivity analysis. Because the short barcode segments themselves contained relatively few informative sites, ML phylogenetic trees were preferentially constructed using IQ-TREE v2 [25]. The optimal nucleotide-substitution model for each dataset was automatically selected by ModelFinder [51] according to the Bayesian information criterion. Branch support was evaluated using 1000 ultrafast bootstrap replicates [52] and 1000 SH-like approximate likelihood-ratio tests [53]. For single-gene trees, branches with UFBoot ≥ 95% and SH-aLRT ≥ 80% were considered strongly supported. Two to three outgroup species closely related to the ingroup were selected according to the current taxonomic system for rooting, and the same outgroups and taxa were used for the short-segment and full-region trees. As a supplementary analysis, NJ trees [54] were also constructed using the Kimura two-parameter distance model [26] and evaluated with 1000 nonparametric bootstrap replicates.

## 5. Conclusions

This study provides a computational, family-level comparison of 31 short candidate segments from 16 nuclear, plastid, and mitochondrial regions for detecting Asteraceae relative to sampled non-Asteraceae Asterales. Regions differed in within-Asteraceae recall, related-family differentiation, primer characteristics, and phylogenetic placement. Conserved markers such as *matR* and *rbcL* showed high target-family identity but limited differentiation; *ITS1*, *ITS*, and *trnH*-*psbA* showed larger background differences; *ITS2* and *ycf1* showed intermediate profiles. These observations are conditional on the dataset, threshold, and search parameters and do not demonstrate causal evolutionary mechanisms, species identification, diagnostic accuracy, or an operational screening. The segments are hypotheses for assay development. Priority next steps are threshold sensitivity analysis, accession- and cluster-audited independent evaluation, congeneric testing, voucher-linked gold standards, prespecified multi-objective selection, and laboratory validation.

## Figures and Tables

**Table 1:** Population genetic parameters of candidate segments from CDS and Non-CDS regions in Asteraceae.

CDSs/Non-CDSs	Genes	*Ps*	Watterson’s *Θ*	*π*	Tajima’s *D*	Number of *Rm*	Density of *Rm* (per bp)
CDSs	*COI (COI-5P)*	0.0283	0.0053	0.0041	−0.7491	1	0.00
CDSs	*matK*	0.4949	0.0682	0.0338	−1.4944	43	0.03
CDSs	*matR*	0.0396	0.0091	0.0056	−1.3759	9	0.00
CDSs	*psbA*	0.1741	0.0240	0.0109	−1.5758	37	0.03
CDSs	*rbcL*	0.3226	0.0444	0.0268	−1.1709	74	0.05
CDSs	*rpoB*	0.3651	0.0503	0.0211	−1.7208	144	0.05
CDSs	*rpoC1*	0.3648	0.0503	0.0214	−1.7062	88	0.04
CDSs	*ycf1*	0.4607	0.0644	0.0375	−1.2479	57	0.01
Non-CDSs	*atpF-atpH*	0.2435	0.0532	0.0351	−1.2130	13	0.02
Non-CDSs	*ITS*	0.5506	0.0600	0.0365	−1.0817	0	0.00
Non-CDSs	*ITS1*	0.3101	0.0505	0.0834	−2.0366	5	0.02
Non-CDSs	*ITS2*	0.5304	0.0531	0.0279	−1.2774	11	0.04
Non-CDSs	*psbK-psbI*	0.2616	0.0485	0.0217	−1.8053	3	0.00
Non-CDSs	*trnL*	0.2170	0.0543	0.0240	−2.1530	6	0.01
Non-CDSs	*trnL-trnF*	0.3150	0.0483	0.0346	−0.8727	0	0.00
Non-CDSs	*trnH-psbA*	0.3908	0.0543	0.0162	−2.0896	2	0.00

**Table 2:** Population genetic parameters of candidate segments from CDS and Non-CDS regions in Asterales.

CDSs/Non-CDSs	Genes	*Ps*	Watterson’s *Θ*	*π*	Tajima’s *D*	Number of *Rm*	Density of *Rm* (per bp)
CDSs	*COI (COI-5P)*	0.2349	0.0379	0.0050	−2.7052	2	0.00
CDSs	*matK*	0.7164	0.0959	0.0632	−1.0033	46	0.03
CDSs	*matR*	0.2745	0.0589	0.0234	−2.1530	47	0.03
CDSs	*psbA*	0.2905	0.0389	0.0103	−2.1537	66	0.06
CDSs	*rbcL*	0.4343	0.0579	0.0399	−0.9124	49	0.03
CDSs	*rpoB*	0.5705	0.0765	0.0422	−1.3215	160	0.05
CDSs	*rpoC1*	0.5651	0.0759	0.0434	−1.2595	120	0.06
CDSs	*ycf1*	0.4954	0.0659	0.0361	−1.3347	17	0.00
Non-CDSs	*atpF-atpH*	0.4348	0.0793	0.0791	−0.0081	7	0.01
Non-CDSs	*ITS*	0.6003	0.0650	0.0336	−1.3295	0	0.00
Non-CDSs	*ITS1*	0.8391	0.1354	0.2214	−1.9810	5	0.01
Non-CDSs	*ITS2*	0.8406	0.0835	0.0464	−1.1887	0	0.00
Non-CDSs	*psbK-psbI*	0.2505	0.0470	0.0148	−2.2958	9	0.02
Non-CDSs	*trnL*	0.4897	0.0561	0.0240	−1.6019	0	0.00
Non-CDSs	*trnL-trnF*	0.4183	0.0485	0.0168	−1.8322	0	0.00
Non-CDSs	*trnH-psbA*	0.5473	0.0696	0.0340	−1.4748	0	0.00

**Table 3:** Descriptive barcode-matching statistics across the four metadata-defined metagenomic sample groups.

Category	Number of Samples (*n*)	Mean Query Cover (%)	Mean Percent Identity (%)
High-confidence target-containing samples (group A)	113	93.17	99.06
Low-abundance or complex-background target-containing samples (group B)	18	90.86	98.41
Non-Asteraceae Asterales background samples (group C)	373	40.45	45.59
Distantly related background samples (group D)	304	10.86	13.02

## Data Availability

This version was consistent with the content of the manuscript: the study analyzed 279,956 locus–record matches retrieved from the NCBI Nucleotide and Gene databases, while the exploratory public metagenomic datasets were obtained from NCBI BioSample and the Sequence Read Archive (SRA). The data-retrieval information and sample metadata were provided in Tables S4 and S5, respectively; the candidate barcode segments and associated computational results were presented in Supplementary Tables S1–S3; the relevant algorithms were described in Supplementary File S1; and the corresponding Python scripts were made publicly available on GitHub (https://github.com/Tetingp/Barcoding1/releases/tag/Barcoding1, accessed on 25 January 2026).

## Supplementary Materials

The following supporting information can be downloaded at: https://www.mdpi.com/article/10.3390/plants15172736/s1, Figure S1: Neighbor-joining phylogenetic trees constructed using the complete sequences of 16 coding and noncoding marker regions from 11 families of Asterales, excluding the candidate barcode segments. Table S1: Nucleotide sequences, conservation scores, and screening scores of the 31 candidate short DNA barcode segments identified from Asteraceae. Table S2: BLASTn matching results and genomic coordinates of the 31 candidate short DNA barcode segments. Table S3: Primer sequences, physicochemical properties, predicted amplicons, and comprehensive in silico specificity results for the 31 candidate short DNA barcode segments. Table S4: Accession numbers, metadata-defined group assignments, sequencing characteristics, and retrieval information for the 808 public metagenomic samples. Table S5: Per-locus NCBI search strategies, accession lists, discovery/test dataset assignments, and sequence, species-level taxon, and genus counts for 16 marker regions across 11 Asterales families. Table S6: Complete pairwise Spearman and Pearson correlation results, effective sample sizes, significance statistics, and uncertainty estimates for the candidate barcode regions under the ANI and AND frameworks. File S1: Detailed algorithms for the mathematical definition of GEDI and OGEDI (Section 4.3), calculating sequence identity and differences associated with target-taxon recall and closely related background discrimination of the barcode segments (Section 4.7), as well as algorithms for calculating the mean differences in the overall geographic distance matrix and overall temporal distance matrix of the collected samples (Section 4.8).

## Abbreviations

AND	Average Nucleotide-Level Difference
ANI	Average Nucleotide-Level Identity
C sites	Conserved sites
CDS	Coding Sequence
GEDI	Genetic Distance
II	Identical pairs
ML	Maximum Likelihood
NCBI	National Center for Biotechnology Information
NJ	Neighbor Joining
Non-CDS	Non-coding Sequence
OGEDI	Overall Genetic Distance
Pi sites	Parsimony-informative sites
Ps	The Proportion of Segregating sites
Rm	Recombination events
S sites	Segregating sites
SI	Transitionsal pairs
SV	Transversional pairs
SVM	Support Vector Machine
V sites	Variable sites
π	Nucleotide Diversity
