# Supplementary material for: Performance Profiles of Short DNA Barcode Segments for Family Level Detection of Asteraceae Within Asterales
Source: Plants (Basel). 2026 Sep 7;15(17):2736. doi: 10.3390/plants15172736 (PMC13568365; doi:10.3390/plants15172736)
Supplement: Supplementary file 1 [file plants-15-02736-s001.zip › Caption of supplementary materials.pdf]

Caption of supplementary materials：

Figure S1: Neighbor-joining phylogenetic trees constructed using the complete sequences of 16 coding and noncoding marker regions from 11 families of Asterales, excluding the candidate barcode segments.

Table S1: Nucleotide sequences, conservation scores, and screening scores of the 31 candidate short DNA barcode segments identified from Asteraceae.

Table S2: BLASTn matching results and genomic coordinates of the 31 candidate short DNA barcode segments.

Table S3: Primer sequences, physicochemical properties, predicted amplicons, and comprehensive in silico specificity results for the 31 candidate short DNA barcode segments.

Table S4: Accession numbers, metadata-defined group assignments, sequencing characteristics, and retrieval information for the 808 public metagenomic samples.

Table S5: Per-locus NCBI search strategies, accession lists, discovery/test dataset assignments, and sequence, species-level taxon, and genus counts for 16 marker regions across 11 Asterales families.

Table S6: Complete pairwise Spearman and Pearson correlation results, effective sample sizes, significance statistics, and uncertainty estimates for the candidate barcode regions under the ANI and AND frameworks.

File S1: Detailed algorithms for mathematical Definition of GEDI and OGEDI (Section 4.3), calculating sequence identity and differences associated with target-taxon recall and closely related background discrimination of the barcode segments (Section 4.7), as well as algorithms for calculating the mean differences in the overall geographic distance matrix and overall temporal distance matrix of the collected samples (Section 4.8).
