# Supplementary material for: Performance Profiles of Short DNA Barcode Segments for Family Level Detection of Asteraceae Within Asterales
Source: Plants (Basel). 2026 Sep 7;15(17):2736. doi: 10.3390/plants15172736 (PMC13568365; doi:10.3390/plants15172736)
Supplement: Supplementary file 1 [file plants-15-02736-s001.zip › Supplementary File S1.pdf]

In 4.3 section. Mathematical Definition of GEDI and OGEDI

For locus (l), the within-family gene evolutionary distance index (GEDI) for family (a) was calculated as:

$$\frac{2}{n_{l,a}(n_{l,a}-1)}\sum_{i=1}^{n_{l,a}-1}\sum_{j=i+1}^{n_{l,a}} d_l(x_{l,a,i}, x_{l,a,j}).$$

For two different families (a) and (b), the between-family GEDI was calculated as:

$$\frac{1}{n_{l,a}n_{l,b}}\sum_{i=1}^{n_{l,a}}\sum_{j=1}^{n_{l,b}} d_l(x_{l,a,i}, x_{l,b,j}), a \neq b.$$

Here, $(n_{l,a})$ and $(n_{l,b})$ denote the numbers of valid sequences from families (a) and (b), respectively; $(x_{l,a,i})$ denotes the (i)-th sequence of family (a) at locus (l); and $(d_l(\cdot,\cdot))$ denotes the pairwise nucleotide distance between two aligned sequences. Thus, $(GEDIl(a,a))$ represents the mean genetic distance within family (a), whereas $(GEDIl(a,b))$ represents the mean genetic distance between families (a) and (b).

OGEDI: Unweighted mean of valid between-family GEDI values for a locus.

In 4.7 section. Equations for Target-Taxon Recall and Discrimination Against Closely Related Background Taxa

The following equations quantify the within-target stability of candidate short barcodes (Identity Calculation) and their ability to discriminate closely related nontarget taxa (Difference Calculation).

1. Definition of Valid Comparison Sites

$$\Omega_j = \{l: q_l \in \{A,C,G,T\},\ r_{jl} \in \{A,C,G,T\}\}$$

2. Number of Valid Comparison Sites

$$V_j = |\Omega_j|$$

3. Nucleotide Identity for an Individual Reference Sequence

$$I_j = \frac{M_j}{V_j} \times 100\%$$

4. Mean Nucleotide Identity

$$\bar{I} = \frac{1}{n_T}\sum_{j=1}^{n_T} I_j$$

5. Proportion of Target Sequences Recovered at Different Identity Thresholds

$$R_I(t) = \frac{\sum_{j=1}^{n_T} \mathbf{1}\left(I_j \geq t\right)}{n_T} \times 100\%$$

6. Proportion of Exact Full-Length Matches

$$F_{\text{exact}} = \frac{\sum_{j=1}^{n_T} \mathbf{1}\left(M_j = L\right)}{n_T} \times 100\%$$

7. Nucleotide Difference for an Individual Reference Sequence

$$D_j = \frac{X_j}{V_j} \times 100\%$$

8. Mean Nucleotide Difference

$$\bar{D} = \frac{1}{n_B}\sum_{j=1}^{n_B} D_j$$

9. Proportion of Nontarget Sequences at Different Difference Thresholds

$$R_D(t) = \frac{\sum_{j=1}^{n_B} \mathbf{1}\left(D_j \geq t\right)}{n_B} \times 100\%$$

10. Proportion Exceeding a Mismatch-Count Threshold

$$R_X(k) = \frac{\sum_{j=1}^{n_B} \mathbf{1}\left(X_j \geq k\right)}{n_B} \times 100\%$$

11. Relationship Between Matches and Mismatches

$$M_j + X_j = V_j$$

12. Relationship Between Identity and Difference

$$I_j + D_j = 100\%$$

Definition of Symbols

Symbol	Definition
q_l	Nucleotide at position l of the candidate short barcode
r_jl	Nucleotide at position l of reference sequence j
Ω_j	Set of valid comparison sites between reference sequence j and the candidate fragment
V_j	Number of valid comparison sites
M_j	Number of matched nucleotides

Symbol	Definition
X_j	Number of mismatched nucleotides
L	Length of the candidate short barcode
n_T	Number of valid reference sequences in the target taxon
n_B	Number of closely related nontarget background sequences
t	Identity or difference threshold
k	Threshold for the number of matched or mismatched nucleotides

Methodological Notes

The metrics above were obtained by sitewise comparison of homologous regions following MAFFT multiple sequence alignment. Identity Calculation assesses the conservation of a candidate short barcode within the target taxon, whereas Difference Calculation assesses sequence divergence between the candidate fragment and closely related nontarget taxa. All proportions were calculated using only valid comparison sites; positions containing N, gaps, or other ambiguous nucleotide codes were excluded from the denominator.

In 4.8 section. Equations for Metric Correlation Analysis

The distance matrices were constructed as follows:

For any two samples i, j, temporal distance was defined as:

$$D_{ij}^{time} = | \; time\_point_i - time\_point_j \; |$$

The unit was **days**. If time_point was missing for either sample, the corresponding pairwise distance was recorded as NA.

For any two samples i, j, the Haversine great-circle distance was calculated using an Earth radius of $R = 6371$ km:

$$D_{ij}^{geo} = 2R\arcsin\sqrt{\sin^2\frac{\Delta\varphi}{2} + \cos(\varphi_i)\cos(\varphi_j)\sin^2\frac{\Delta\lambda}{2}}$$

If geographic coordinates were missing for either sample, the corresponding pairwise distance was recorded as NA.

Values in the distance matrices were then summarized as follows:

Mean Pairwise Geographic Distance (mean_geo_km)

Let the geographic distance matrix be D^{geo}. All valid entries in its upper triangle, excluding the diagonal, were retained:

$$\bar{D^{geo}} = \frac{1}{m} \sum_{i<j,\, D_{ij}^{geo} \neq NA} D_{ij}^{geo}$$

where m is the number of valid sample pairs, corresponding to n_geo_pairs_used in the output table.

Mean Pairwise Temporal Distance (mean_time_days)

Similarly, for the temporal distance matrix D^{time}:

$$\bar{D^{time}} = \frac{1}{m'} \sum_{i<j,\, D_{ij}^{time} \neq NA} D_{ij}^{time}$$

where m' is the number of valid sample pairs, corresponding to n_time_pairs_used in the output table.

Noting:
Mean pairwise geographic and temporal distances were calculated as the averages of all nonmissing values in the upper triangles of the respective distance matrices, excluding diagonal elements.
